# Chromosome position effects on gene expression in *Escherichia coli* K-12

**DOI:** 10.1093/nar/gku828

**Published:** 2014-09-10

**Authors:** Jack A. Bryant, Laura E. Sellars, Stephen J. W. Busby, David J. Lee

**Affiliations:** Institute of Microbiology and Infection, School of Biosciences, University of Birmingham, Edgbaston, Birmingham, B15 2TT, UK

## Abstract

In eukaryotes, the location of a gene on the chromosome is known to affect its expression, but such position effects are poorly understood in bacteria. Here, using *Escherichia coli* K-12, we demonstrate that expression of a reporter gene cassette, comprised of the model *E. coli lac* promoter driving expression of *gfp*, varies by ∼300-fold depending on its precise position on the chromosome. At some positions, expression was more than 3-fold higher than at the natural *lac* promoter locus, whereas at several other locations, the reporter cassette was completely silenced: effectively overriding local *lac* promoter control. These effects were not due to differences in gene copy number, caused by partially replicated genomes. Rather, the differences in gene expression occur predominantly at the level of transcription and are mediated by several different features that are involved in chromosome organization. Taken together, our findings identify a tier of gene regulation above local promoter control and highlight the importance of chromosome position effects on gene expression profiles in bacteria.

## INTRODUCTION

The nucleoid is a highly compact and organized structure occupying the majority of the intracellular cytoplasmic space in most bacteria (1,2). Comprised of chromosomal DNA, protein and RNA, the nucleoid in *Escherichia coli* and *Salmonella* is arranged into topologically isolated loops, each ∼10 kb in length, which are further organized into four spatially isolated, structured macrodomains and two non-structured regions (3–5). Organization of the nucleoid is mediated by DNA supercoiling, macromolecular crowding and by a number of nucleoid associated proteins (NAPs), although the precise impact of each on the overall structure is not fully understood. All NAPs influence DNA conformation: with some binding predominantly within one macrodomain and others binding ubiquitously throughout the genome (6–9). Highly proteinaceous transcriptionally silent regions of the chromosome have been identified as potential organizational hubs that may insulate the topologically isolated loops and macrodomains (10). Termed transcriptionally silent Extended Protein Occupancy Domains (tsEPODs), these domains overlap with regions bound by NAPs, but neither the precise protein organization nor whether tsEPODs contain predominantly poor promoters, or active promoters silenced by the associated proteins, is known.

Despite being highly compacted, the nucleoid remains accessible for cellular processes such as transcription and replication. Transcriptionally active regions are thought to be extruded to the periphery of the nucleoid where they are engaged by transcription foci, dense in RNA polymerase (RNAP) (11–17). These foci are located in discrete areas of the cell, indicating that some transcription events occur at particular locations and that the specific chromosomal position and spatial organization of genes may be important for maintaining control of gene expression (13,16). In eukaryotic systems, it is well established that the expression of individual genes can be greatly affected by chromosomal position (18–21). Most recently, Akhtar *et al.* (22) analyzed ∼27 000 reporter gene integrations in mouse embryonic stem cells and demonstrated that expression varied across the genome by more than 1000-fold. Furthermore, expression levels were reflective of the local chromosomal environment: being attenuated in lamina binding domains and areas of compaction, and enhanced when located proximal to active genes.

Only a handful of similar studies have been conducted in bacterial systems, with all attributing minor differences in gene expression to gene dosage effects: the correlation between the increase in gene expression and the proximity of the gene to the origin of replication (23–31). Here we have re-addressed chromosomal position effects in *E. coli* by inserting a transcription reporter cassette at different targeted positions in the genome. We observed substantial position-dependent variation of promoter activity that is mediated at the point of transcription and is unrelated to gene dosage. We identified several factors that impact upon gene expression, including processes that are involved with chromosome structuring and organization.

## MATERIALS AND METHODS

### Strains and plasmids

Bacterial strains and plasmids used in this study are listed in Supplementary Table S1. The position of the *lac* promoter::*gfp* insertion site is given in base pairs with respect to the coordinate system origin (32).

### Construction of targeted recombineering plasmids

The reporter cassette was constructed in plasmid pKH5 (33), by replacing the *lacI* homology region with a multiple cloning site (MCS 1) and the *lacZ* homology region with the Emerald *gfp* gene (Invitrogen) and a multiple cloning site (MCS 2) (Supplementary Figure S1). The *lac* promoter, from position −93 to +122 bp, relative to the *lacZ* transcription start site, was amplified by polymerase chain reaction (PCR) from *E. coli* K-12 genomic DNA using primers D68498 and D69482, and cloned into the reporter cassette construct upstream of the Emerald *gfp* gene. The reporter cassette, flanked by *I-SceI* restriction sites, was cloned into *I-SceI* digested pDOC-C (34), generating plasmid pJB (Supplementary Figures S1 and S2). To target the reporter cassette to the chromosome of *E. coli* K-12 MG1655, ∼500 bp regions homologous to chromosomal targets were amplified by PCR and cloned into MCS 1 and MCS 2 in plasmid pJB. Oligonucleotides used for cloning are listed in Supplementary Table S2.

### Chromosomal recombination

The pJB donor plasmids, carrying homology to the chromosome, were used to transfer the *lac* promoter*::gfp* fusion to the specifically targeted chromosomal loci (Supplementary Figures S3–S5). *E. coli* K-12 MG1655 was co-transformed with a pJB donor plasmid and plasmid pACBSCE, after which the donor fragment was integrated into the chromosome, using the gene doctoring method (34). Recombinants were screened for the presence of the insert by colony PCR and targeted insertion strains were assigned a BRY strain number (Supplementary Table S1). The kanamycin resistance cassette was excised from the chromosome using flippase (FLP) recombinase, expressed from plasmid pCP20 (35). Candidates were re-screened by colony PCR to confirm *kan*^R^ gene removal.

### Fluorescence assays

Bacterial cultures were grown for 16 h at 37°C with aeration in M9 minimal salts media, supplemented with 0.3% fructose, 2 mM MgSO_4_, 0.1 mM CaCl_2_ and 0.1% casamino acids. Cultures were diluted 100-fold into 5 ml of fresh medium to a starting OD_620_ of ∼0.03. Additional supplements were added where stated in figure legends. Cultures were incubated at 37°C with aeration until an OD_620_ of 0.4–0.5 was reached. At this point, 250 μl samples of each culture were aliquoted into a sterile, black, optically clear 96-well Corning Costar 3603 plate (Thermo Scientific). Fluorescence at excitation wavelength 485 nm and emission wavelength 510 nm was measured for an integration time of 1 s using a Thermo Fluoroskan Ascent FL fluorometer (Thermo Scientific) after a 10 s shake step at 600 rpm. Each experiment consisted of a minimum of three biological replicates and experiments were repeated at least twice. As a control, readings were taken from *E. coli* K-12 MG1655 cultures, for which, no fluorescence was detected. Fluorescence output from the reporter cassette was derived as fluorescence/OD_620_ to represent specific fluorescence of the culture, with mean and standard deviation calculated for each strain/condition.

### Gene dosage measurements

Quantitative real-time PCR (qRT-PCR) was used to measure the amount of *gfp* gene in different BRY strains. Genomic DNA was extracted using the illustra bacteria genomic Prep Mini Spin Kit (GE Healthcare). The DNA concentration was determined using a NanoDrop ND-1000 spectrophotometer (Thermo Scientific). gDNA was used as template in qRT-PCR using an Mx3000P qPCR system (Agilent) and Brilliant III Ultra-Fast SYBR Green QPCR master mix (Agilent). Oligonucleotides designed for detection of the *gfp* gene and the internal control, *bglB*, are listed in Table S2. Relative quantities of the *gfp* target gene were determined by normalizing reaction threshold cycle (*C*_T_) values to that of the *bglB* reference gene. Δ*C*_T_ values for the BRY33 reactions were used as calibrators (ΔΔ*C*_T_ = Δ*C*_T target_ – Δ*C*_T BRY33_) for analysis of results by the relative quantification method (2^−ΔΔ*C*^T), which was used with standard curves (36). Quantities of the *gfp* target gene, relative to that at the *tam* locus, are represented as gene dosage. Each reaction was repeated at least three times for each of three separate biological replicates to yield mean and standard deviation for each experiment.

### Chromatin immunoprecipitation and qPCR analysis

Chromatin immunoprecipitation followed by qPCR was used to quantify the amount of RNAP occupancy within the *gfp* gene in different BRY strains. ChIP-qPCR was done as described previously, using antibody raised against the RNAP β subunit (Neoclone # W0002) (37). The oligonucleotides used to amplify the *gfp* gene target are listed in Supplementary Table S2.

### RNA isolation and qRT-PCR analysis

qRT-PCR was used to quantify the relative expression levels of *gfp* mRNA in different BRY strains. Total RNA was isolated using RNA later (Ambion) stabilization solution and an RNeasy Mini kit with on-column DNase I digestion (Qiagen). Total RNA was reverse transcribed using a tetro cDNA synthesis kit (Bioline) with oligonucleotides specific for the *bglB* and *gfp* genes (Supplementary Table S2). cDNA was used as template in qPCR using an Mx3000P qPCR system (Agilent) and Brilliant III Ultra-Fast SYBR Green QPCR master mix (Agilent). Relative expression levels of the *gfp* target gene were determined by normalizing reaction threshold cycle (*C*_T_) values to that of the *bglB* reference gene. Δ*C*_T_ values for un-induced, no isopropyl β-D-1-thiogalactopyranoside (IPTG), cultures were used as calibrators for analysis of results by the relative quantification method (2^−ΔΔ*C*^T) (36). Each reaction was repeated three times for each of three separate biological replicates to yield mean and standard deviation values.

### Chloroquine agarose gel electrophoresis

BRY35 cells carrying plasmid pBR322 were grown in minimal media supplemented with ampicillin (80 μg/ml) and different concentrations of novobiocin (0–100 μg/ml). Plasmid DNA was purified using a QIAprep Spin Miniprep Kit (Qiagen) and topoisomers were resolved by 1% agarose gel electrophoresis, supplemented with 2.5 μg/ml chloroquine in 2% tris-borate-EDTA (TBE) buffer, for 24 h at 3 V/cm in the dark. Chloroquine was removed from the gel by rinsing with distilled water for 2 h after which the gel was stained with ethidium bromide and visualized under UV light.

### α-galactosidase assay

To eliminate interference of melibiose transport by the *lacY* encoded Lac permease (38), we deleted the wild-type *lac* promoter in strains BRY15 and BRY37, using the method described by Hollands (33). The resulting BRY75 and BRY79 strains were grown for 16 h with aeration in 5 ml M9 minimal salts media at 30°C. Cultures were diluted 100-fold into 5 ml of fresh medium to a starting OD_650_ of ∼0.03. Cultures were supplemented with 100 μM IPTG or 0.2% melibiose, to induce *melAB* transcription, where required. Cultures were incubated at 30°C with aeration until mid-logarithmic phase of growth and the OD_650_ of the culture recorded. 50 μg/ml chloramphenicol was added to each culture to arrest protein production. 4-nitrophenyl-d-galactopyranoside (PNPG) was added to each culture to a final concentration of 3 mM, and incubated at 30°C with aeration until a yellow colour developed, after which the reaction was stopped by addition of ethylenediaminetetraacetic acid and Na_2_CO_3_ to final concentrations of 40 and 250 mM, respectively. The OD_410_ of the reaction was recorded and α-galactosidase activities calculated as follows: α-galactosidase activity = (2.5 × V × ΔOD_410_)/(*t* × 0.0045 × 2 × OD_650_) where: 2.5 = factor for conversion of OD_650_ into bacterial mass, based on OD_650_ of 1 being equivalent to 0.4 mg/ml bacteria (dry weight); V = final assay volume (ml); 0.0045 = factor for conversion of OD_410_ into nmol *p*-nitrophenyl (PNP), based on 1 nmol ml^−1^ PNP having an OD_410_ of 0.0045; *t* = incubation time (min); v = volume of culture added (in ml) (39). Calculated α-galactosidase activity is therefore represented as nmoles PNP liberated/min/mg bacteria. Data are presented from a minimum of three biological replicates for each strain tested, repeated on at least two separate occasions.

## RESULTS

### Chromosomal position affects gene expression

**Figure 1.**
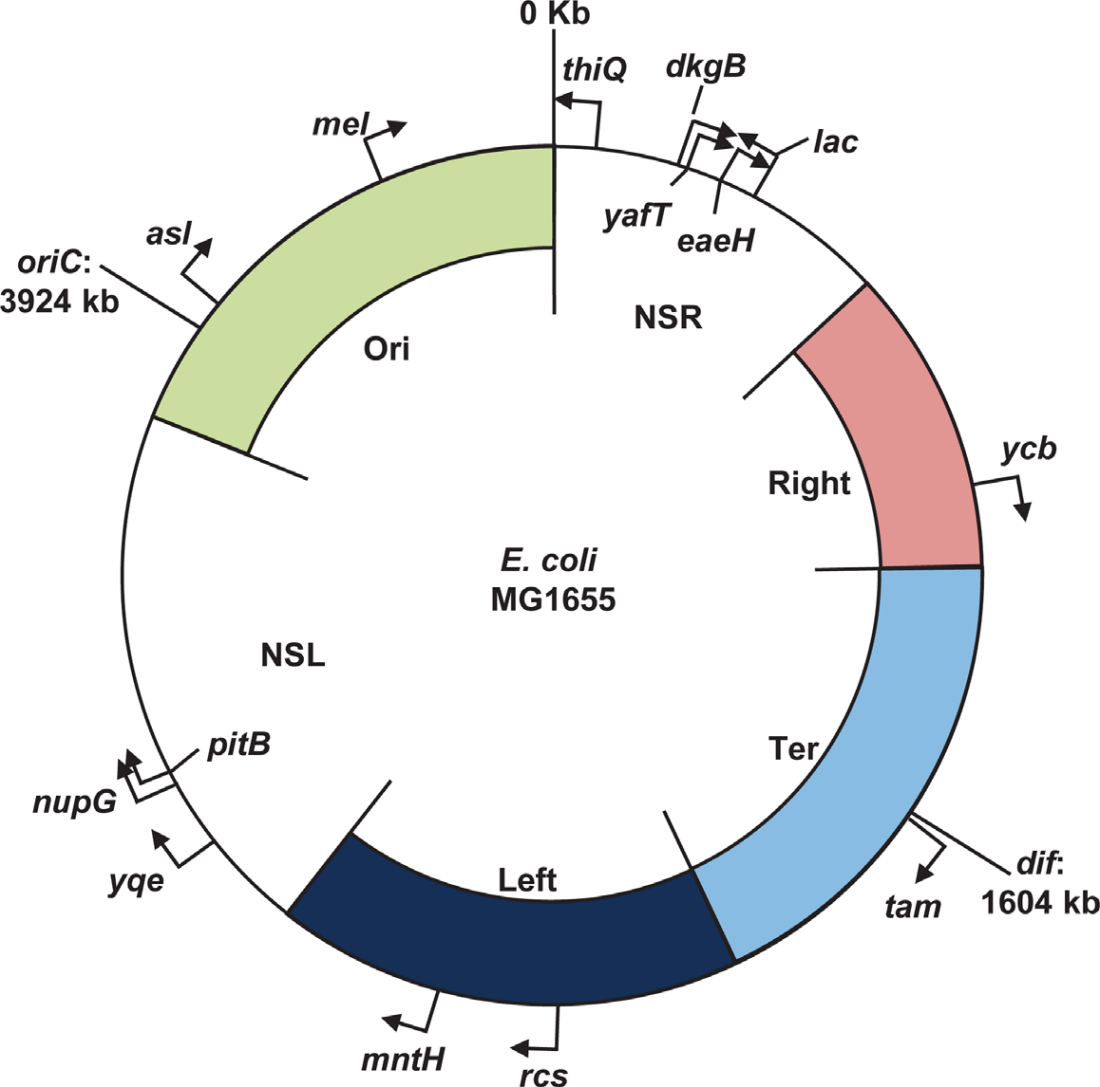
Insertion of the reporter cassette into the *E. coli* K-12 MG1655 genome. The chromosome positions of *oriC* and *dif* labelled, relative to the coordinate system origin (32). The structured macrodomains, Ori, Right, Ter and Left, are represented as coloured arcs with macrodomain boundaries shown inside the circular map (4). The non-structured left and right regions are labelled as NSL and NSR respectively. The orientation of the reporter cassette at the chromosome insertion sites is indicated by a black arrow.

**Figure 2.**
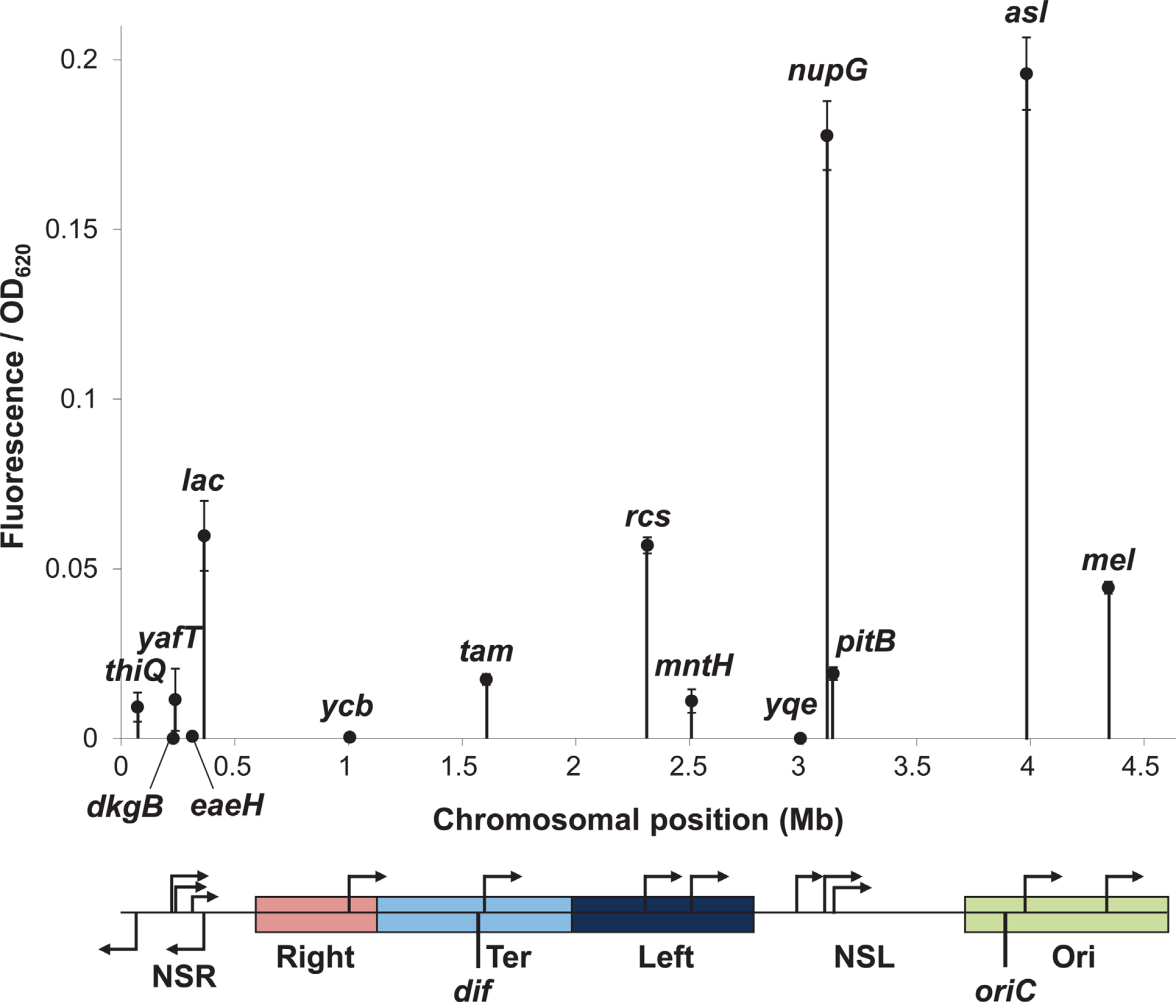
Effect of chromosomal position on reporter cassette expression. Fluorescence output from the reporter cassette was measured during growth in the presence of 100 μM IPTG and is represented on the y-axis as a function of OD_620_. In the absence of IPTG no fluorescence was detected. The location and orientations of each reporter cassette insertion site is indicated on the x-axis and on the linear schematic of the genome below. The locations of the macrodomains and non-structured regions (NSR, Right, Ter, Left, NSL and Ori) and the origin (*oriC*) and terminus (*dif*) of replication are also indicated.

To examine the impact of chromosomal position effects on gene expression in *E. coli* we designed a transcription reporter cassette that expressed a measurable readout. The cassette consisted of the *E. coli lac* promoter controlling production of Emerald GFP: hence, expression of *gfp* was triggered by addition of IPTG. The cassette was insulated from transcription read-through by the bacteriophage lambda *oop* terminator (33,40), located upstream of the *lac* promoter (Figure 1A). Emerald GFP was chosen because the gene has a higher GC bp content and is predicted to contain less DNA curvature, thus limiting undesirable associations with NAPs that favour AT bp rich, curved DNA (Supplementary Figure S6) (41). To avoid impacting upon local gene regulatory elements, the cassette was deliberately targeted to non-coding regions between convergent genes at different chromosomal positions, within each of the four macrodomains and two non-structured regions, in the *E. coli* K-12 strain, MG1655 (Figure 1B). Fourteen insertion positions were selected in the MG1655 genome, which were named based on the neighbouring gene (Figure 1 and Supplementary Figures S3–S5). GFP fluorescence measurements were taken during logarithmic growth in minimal media, supplemented with IPTG, and we observed that chromosomal position modulates gene expression from the reporter cassette over a ∼300-fold range (Figure 2). Compared to insertion at the wild-type *lac* locus, GFP fluorescence was more than 3-fold higher at the *nupG* and *asl* loci and lower at every other insertion position. Expression levels varied between each of the macrodomains and expression within each macrodomain fluctuated by 5-fold (in Left and Ori), 10-fold (in the non-structured left region) or 100-fold (in the non-structured right region).

### Chromosomal position effects are not solely due to gene dosage

**Figure 3.**
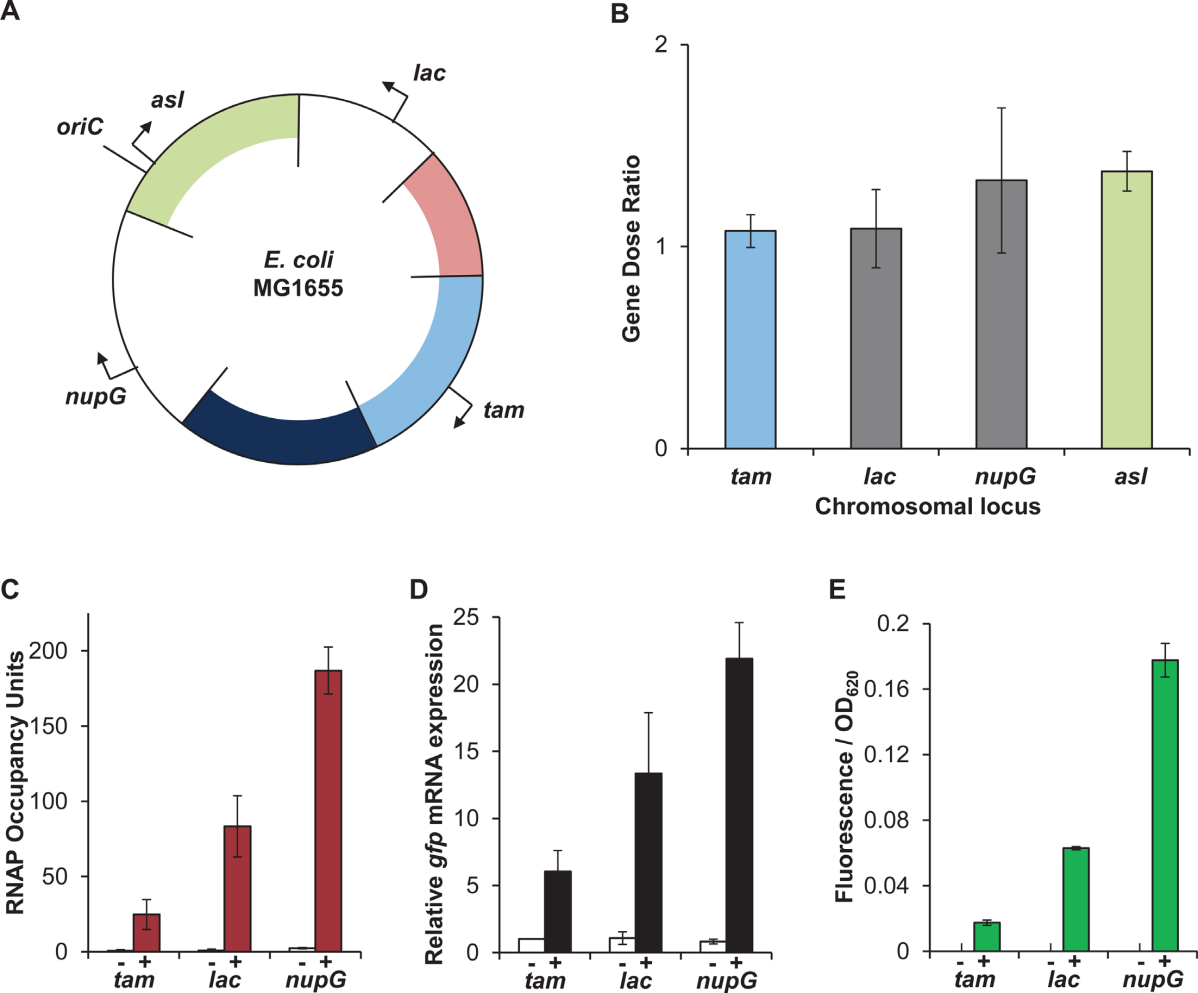
Position effects occur at the level of transcription. (**A**) Circular map of the *E.coli* MG1655 chromosome with the position of the reporter cassette at the *tam*, *nupG, lac* and *asl* positions marked. (**B**) Gene dose ratio of the *gfp* gene at the *tam*, *lac* and *nupG* loci, relative to the *tam* locus. (**C**) RNAP occupancy within the *gfp* gene, at the *tam*, *lac* and *nupG* loci, measured by ChIP-qPCR. (**D**) *gfp* mRNA expression upon induction of the reporter cassette at the *tam*, *lac* and *nupG* loci (Data are normalized to the un-induced *tam* culture). (**E**) Fluorescence output from the reporter cassette at the *tam*, *lac* and *nupG* loci. (C–E) Cells were grown with the inducer of the *lac* operon, IPTG (100 μM: filled bars) or without (white bars), denoted as + or − below the x-axis.

To substantiate our observations, we conducted control experiments to confirm that variation in expression from the reporter cassette was solely due to local chromosomal position effects. Previous analyses of these effects in bacteria have concluded that variations observed in expression upon gene translocation are minimal and are predominantly due to gene dosage (23–27). Thus, to define the consequence of gene dosage on *gfp* expression in our system we determined the number of DNA copies of the reporter at four different genomic loci (Figure 3A). Total genomic DNA was isolated from strains carrying the reporter cassette at the *tam, lac, nupG* and *asl* loci and the relative amounts of the *gfp* gene were determined by qPCR. The relative copy number of the *gfp* gene varied by only 1.4-fold between the different loci and as expected, the biggest variation occurred between *oriC* (*asl*) and ter (*tam*) proximal targets (Figure 3B). Therefore, gene dosage can only account for 1.4-fold differences in position-dependent variation of gene expression in the experiments reported here.

### Chromosomal position effects are mediated at the level of transcription

Since the gene copy number did not correlate with the differences observed in expression across the genome, we considered the possible impact of transcription events originating from elsewhere within the reporter cassette or from the chromosome adjacent to the cassette. We also considered the possible impact of post-transcriptional processes. First, we assessed transcription directly by measuring RNAP occupancy within the *gfp* gene located at the *tam, lac* and *nupG* loci in the presence and absence of the inducer of the reporter cassette, IPTG. To do this we used *ch*romatin *i*mmuno*p*recipitation (ChIP) with antibodies against the β subunit of RNAP and quantified the amount of immunoprecipitated *gfp* DNA by quantitative PCR. The results show that RNAP occupancy of the *gfp* gene correlates well with the fluorescence output at the three loci (Figure 3C and E). Importantly, RNAP was not observed within the *gfp* gene in the absence of the inducer IPTG. This demonstrates that occupancy of the *gfp* gene, and therefore differences in fluorescence, are due to different levels of transcription of the *gfp* gene derived only from the *lac* promoter within the reporter cassette, and not from transcriptional read-through from neighbouring genes or from transcription originating from elsewhere within the reporter cassette.

We next measured the amount of *gfp* mRNA transcript after isolation of total RNA from the three different strains. We observed that the relative levels of transcript from the three loci did not fully correlate with the amount of RNAP occupancy (Figure 3C and D), which could indicate that there are differences in mRNA stability or access to ribosomes at the different chromosome loci. However, as we only observed transcription of *gfp* in the presence of IPTG, which correlates well with fluorescence output, we suggest that the level of transcription is the predominant contributing factor which sets the level of gene expression from each loci, with minor fluctuations in mRNA stability and rates of translation accounting for minimal variation.

### Low expression is due to silencing in tsEPODs

**Figure 4.**
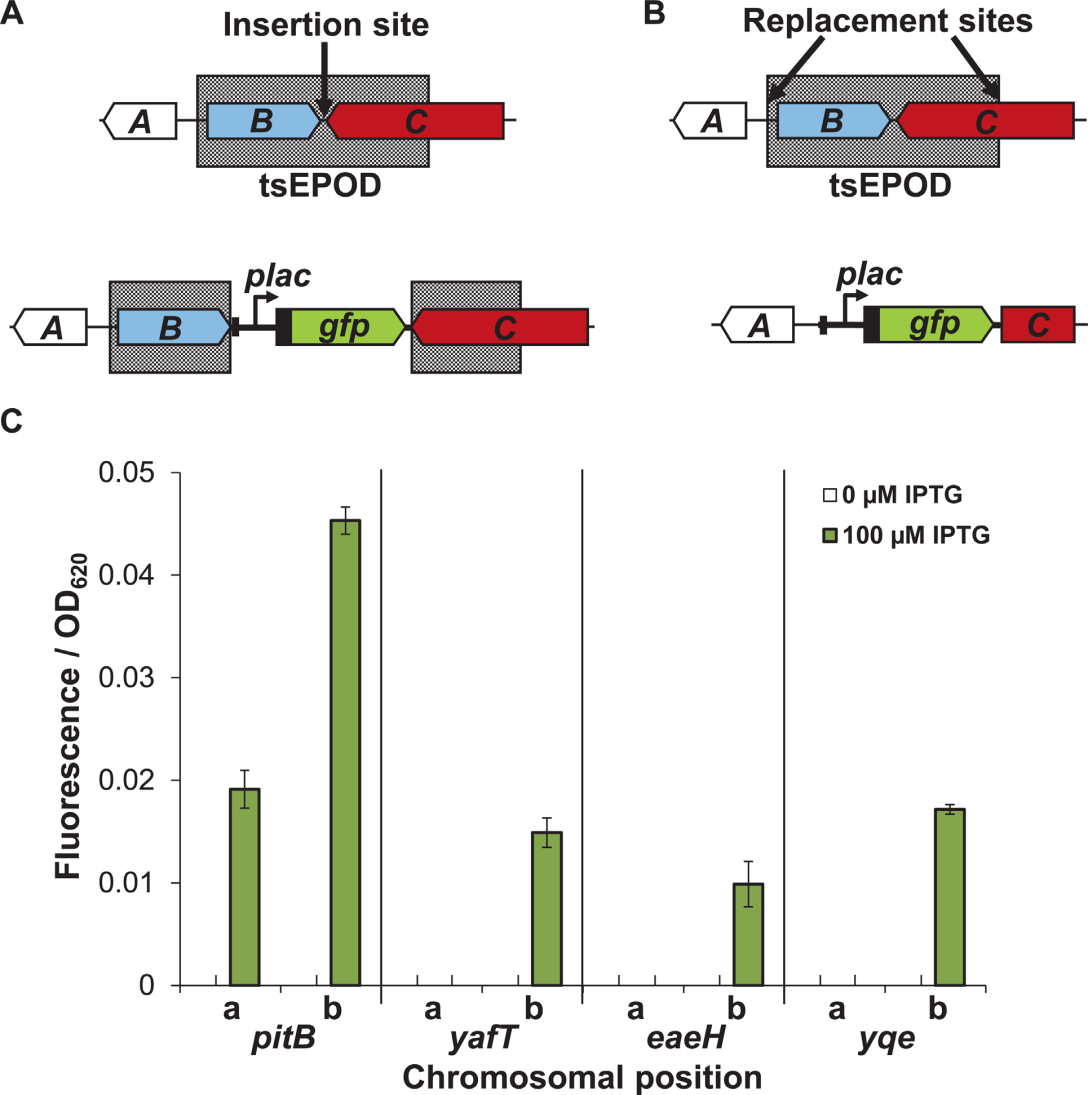
tsEPODs silence activity of the *lac* promoter. (**A**) Example of insertion of the reporter cassette within a tsEPOD (grey box). Genes are represented as block arrows, labelled *A, B* and *C*. (**B**) Replacement of tsEPOD sequence with the reporter cassette. (**C**) Fluorescence output from the reporter cassette from the *pitB, yafT, eaeH*, *yqe* loci. On the x-axis, **a** or **b** denotes tsEPOD disruption or replacement respectively.

To understand better the mechanisms that caused variation in reporter cassette activity across the genome, we considered several chromosomal features and their impact on gene expression. At the *yafT*,*eaeH*,*yqe* and *pitB* loci, the reporter cassette was inserted into a tsEPOD. Activity of the reporter cassette was significantly reduced in each case, indicating that transcription of the active *lac* promoter was suppressed by the tsEPOD (Figure 2). To assess whether suppression was directly due to intrinsic properties of the tsEPODs, we re-introduced the reporter cassette at the *yafT*,*eaeH*,*yqe* and *pitB* loci by replacing the tsEPOD, rather than inserting within (Figure 4A and B and Supplementary Figures S4 and S5). GFP fluorescence of each tsEPOD replacement strain was then compared to the tsEPOD insertion strains and, in all cases, replacement resulted in de-repression of the reporter cassette (Figure 4C). It was noted that although the tsEPODs tested were capable of silencing the reporter cassette, both the extent of silencing and of de-repression after EPOD replacement varied, suggesting that additional local chromosomal features may modulate gene expression at these loci.

### DNA gyrase plays a role at high activity locations

**Figure 5.**
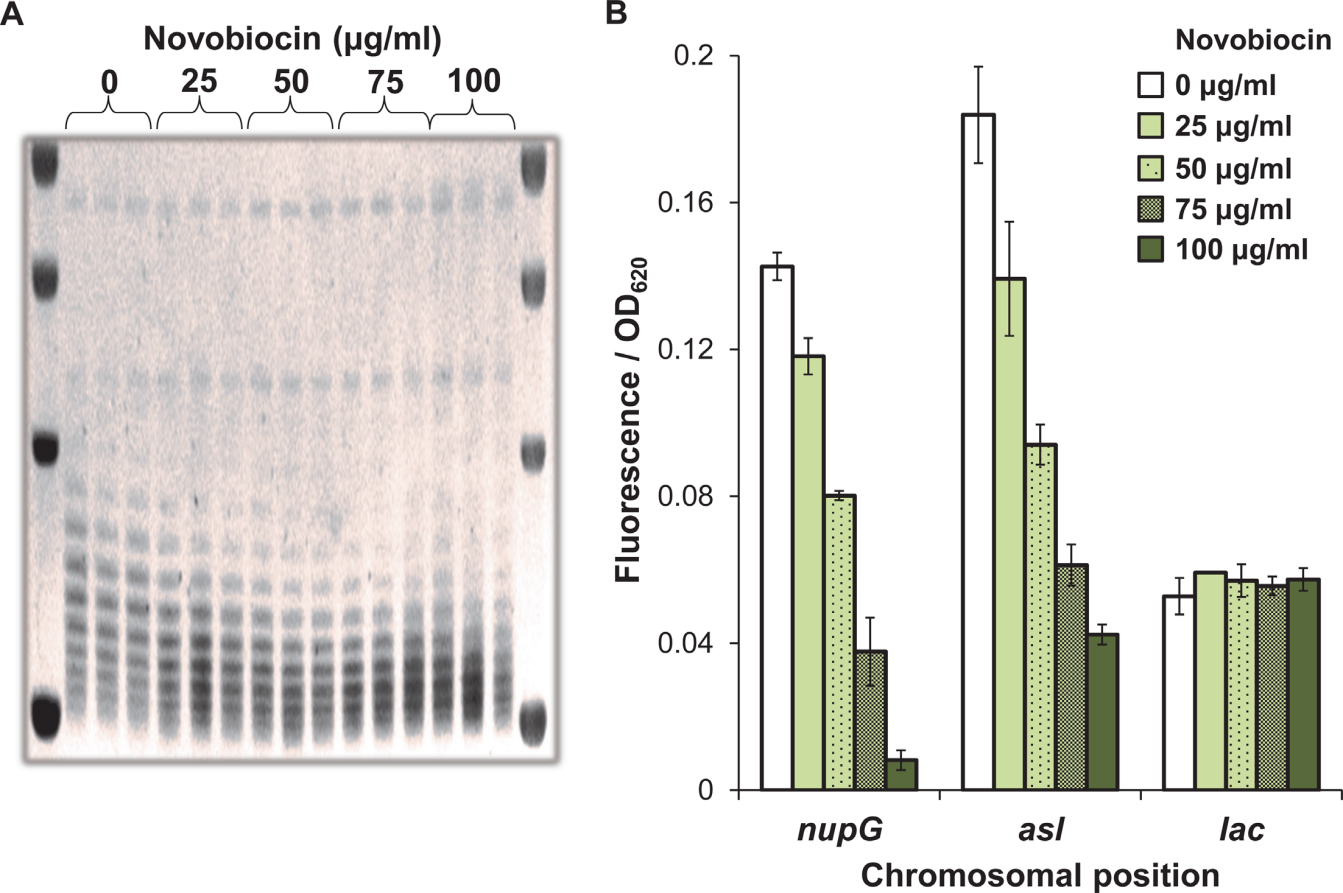
DNA gyrase influences expression at high activity insertion sites. (**A**) pBR322 plasmid was used as a reporter of DNA superhelicity during growth with increasing concentrations of novobiocin. Plasmid DNA was separated on a 1% agarose gel supplemented with 2.5 μg/ml chloroquine. Ethidium bromide stained DNA was visualized under UV light. (**B**) Fluorescence output from the reporter cassette at the *nupG*, *asl* and *lac* positions, during growth in the presence of 100 μM IPTG and increasing concentrations of novobiocin.

The impact of nucleoid topology on gene expression was assessed by analyzing the effects of novobiocin addition: an inhibitor of the GyrB subunit of *E. coli* DNA gyrase, which is solely responsible for introducing negative supercoils into the genome (42). To evaluate gyrase inhibition, the degree of supercoiling of plasmid pBR322 was assessed over a range of novobiocin concentrations. Plasmids were harvested and visualized by chloroquine agarose gel electrophoresis, to separate the different supercoiled plasmid topoisomers. Changes in migration of pBR322 confirmed that superhelicity is shifted to a less negative state with increasing concentrations of novobiocin in the growth medium (Figure 5A). These concentrations were then used to assess the impact of supercoiling on expression from the reporter cassette and it was observed that only the high activity loci, *nupG* and *asl* were affected. Inhibition of GyrB reduced reporter cassette expression at the *nupG* locus by 17-fold, and at the *asl* locus by 4-fold (Figure 5B). In the presence of a sub-inhibitory to growth concentration of novobiocin (50 μg/ml), reporter cassette activity was ∼2-fold reduced at both the *nupG* and *asl* loci. This resulted in expression levels comparable to the *lac* locus, suggesting that the high promoter activity at the *nupG* and *asl* loci is largely due to the action of DNA gyrase.

### Neighbouring gene expression influences downstream promoters

**Figure 6.**
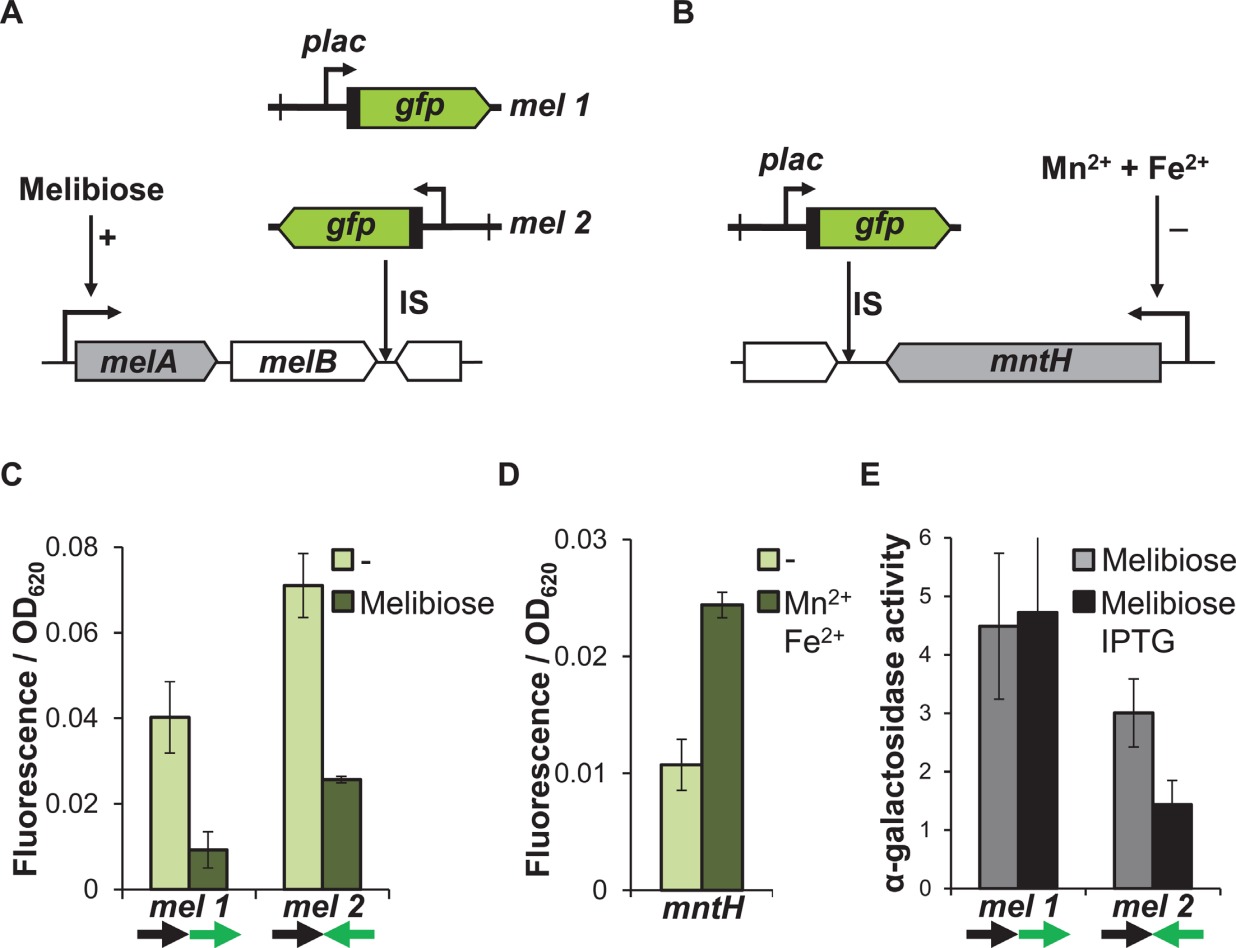
Active transcription has a negative effect on downstream promoters. (**A**) Schematic representation of gene organization at the *mel* locus. (**B**) Schematic representation of gene organization at the *mntH* locus. (**C**) Fluorescent output from the reporter cassette at the *mel* locus during growth with 100 μM IPTG, in the presence or absence of the inducer of the *melAB* operon, melibiose (0.2%). (**D**) Fluorescence output from the reporter at the *mntH* locus during growth with 100 μM IPTG, in the presence or absence of 10 μM MnCl_2_ and 10 μM (NH_4_)_2_Fe(SO)_4_. (**E**) α-galactosidase activity measured in strains with the reporter cassette inserted at the *mel* locus during growth with 100 μM IPTG or 0.2% melibiose. Arrows indicate co-directional (→→) or head to head (→←) transcription.

Insertion of the reporter cassette at the *mel* and *mntH* positions provided the opportunity to measure the effects of neighbouring gene expression, since expression of *mntH* and the *melAB* operon could be specifically controlled (Figure 6A and B) (38,43). Thus, expression of the reporter cassette could be measured when expression of the upstream neighbouring gene was on or off. At the *melAB* locus, induction of the upstream *melAB* operon by the addition of melibiose resulted in a reduction of downstream reporter cassette expression by 4-fold, regardless of its orientation (Figure 6C). A similar effect was observed at the *mntH* locus where, upon repression of the upstream *mntH* promoter by the addition of Mn^2+^ and Fe^2+^ ions, activity of the downstream reporter cassette increased 3-fold (Figure 6D). To examine this further, we measured the impact of reporter cassette transcription on *melAB* expression, by assaying the activity of the α-galactosidase enzyme, encoded by *melA*. When transcription from the reporter cassette was directed away from the *melAB* operon, α-galactosidase activity was unaffected by induction of the reporter cassette. However, induction of reporter cassette transcription towards the *melAB* operon resulted in a 50% reduction in α-galactosidase activity (Figure 6E), indicating that transcription events only repress expression of downstream neighbouring genes, irrespective of the orientation of the downstream gene.

## DISCUSSION

Few studies have directly assessed the effect of position within bacterial chromosomes on gene expression, with only small effects reported that were attributed to gene dosage (23–31). Here, by growing bacteria in minimal nutrient medium, we limited variations in gene dosage to a maximum of 1.4-fold across the genome. To minimize disruption to local chromosomal processes, we deliberately targeted non-coding, non-regulatory elements of the genome with a discrete reporter cassette that was small compared to previously used promoter::reporter probes (23,26,27). We show that gene expression varies between insertion sites within the same macrodomain, and that macrodomains and non-structured regions contain both high and low activity regions. In addition, we demonstrated that position-dependent variation in output from the reporter cassette was solely due to transcription of *gfp* derived from the *lac* promoter. Therefore, our observation that position-dependent gene expression levels can vary by ∼300-fold indicates that substantial differences in expression potential exist within bacterial genomes.

Concerning silencing, previously Vora *et al.* (10) identified 151 tsEPODs distributed throughout the *E. coli* genome, which had an average length of 2050 bp. The genomic positions of the tsEPODs were found to correlate with regions of the chromosome that have high NAP occupancy, as determined by ChIP analysis, and thus were proposed to act as nucleoid organizational hubs. Insertion of the reporter cassette within tsEPODs resulted in substantial suppression of promoter activity, which was only restored when the tsEPOD was replaced by the reporter cassette, suggesting that theses domains are capable of silencing transcription. It is not known whether silencing within tsEPODs is due to the binding of one particular NAP, a combination of NAPs binding, or the associated DNA architecture. What is clear is that transcription is repressed by the intrinsic properties of the tsEPOD, as opposed to the tsEPOD merely containing poor promoters. This is reminiscent of lamina-associated domains in eukaryotic systems. Lamins organize chromatin by interactions with Lamina-associated domains that typically span several megabases of DNA: much larger than tsEPODs. Similarly to tsEPODs, lamina-associated domains are typified by low gene expression levels, which have been shown to confer low activity upon inserted reporter cassettes (21,22,44). However, in contrast to tsEPODs, which are predicted to be buried inside the nucleoid, Lamina-associated domains are at the periphery of the nucleus, anchored to the nuclear membrane.

Several other loci were identified where expression of the reporter cassette was silenced, which were not located within tsEPODs. However, at the *ycb* locus, the reporter cassette was inserted within 500 bp of a tsEPOD, which may influence the expression from this target. At the *dkgB* locus, the reporter cassette was inserted immediately downstream of a ribosomal operon encoding a ribosomal RNA and a tRNA, which are likely to be highly expressed. We therefore suggest that transcription of the reporter cassette is repressed as a consequence of high levels of neighbouring transcription. The nature of the silencing effect at the *thiQ* locus is not known: this insertion locus is neither in a tsEPOD or neighbouring a highly expressed gene.

Examination of the high expression levels at the *asl and nupG* loci determined that activity was dependent upon the DNA supercoiling activity of DNA gyrase. Inhibition of gyrase severely impaired reporter cassette expression at the *asl* and *nupG* loci, but had little or no affect at other locations. The genome-wide DNA gyrase distribution was previously determined by analyzing DNA association by ChIP-chip experiments (45,46). The resolution of these experiments was insufficient to enable identification of specific DNA gyrase binding sites, but the data clearly demonstrated an increasing density gradient of DNA gyrase binding sites proximal to the origin of replication. Correlations have been drawn between the close proximity of highly expressed ribosomal operons to the replication origin and their high-dependency on DNA gyrase induced negative superhelicity (46,47). However, we observed low expression from the reporter cassette inserted at the *yqe* and *pitB* loci, immediately adjacent to the *nupG* locus, and at the *thiQ* and *dkgB* loci, suggesting that proximity to the *ori* does not intrinsically result in high expression due to the activity of DNA gyrase.

Transcription of the reporter cassette was found to have a profound impact on expression of neighbouring genes. When transcription was directed towards a transcription unit the activity of the downstream transcription unit was repressed, irrespective of orientation. These effects may be due to diffusion of transcription induced positive supercoiling created ahead of RNAP, which impacts upon the ability of the downstream RNAP to transcribe (48,49). This phenomenon is described as the twin-supercoiling domain model and accounts for a large quantity of DNA supercoiling within the bacterial cell (48,50–52). Such supercoiling can diffuse along the DNA to affect local chromosome structures several kilobases away from the site of transcription and is dependent upon promoter strength (49,53,54).

In conclusion, we have identified several mechanisms that account for the variations in gene expression that we observed, but they are by no means all encompassing. For example, we see silencing of gene expression at tsEPODs but it is not clear which NAPs are bound at a particular tsEPOD and how they interplay to silence transcription. Hence, further scrutiny of the spatial and temporal dynamics of the nucleoid, and the mechanisms that we have identified, is essential to appreciate the full impact of chromosomal position effects in bacteria. However, our findings do clearly demonstrate profound differences in gene expression due to chromosomal location and hence, verify position effects as a *bona fide* gene regulatory feature of bacterial genomes.

## SUPPLEMENTARY DATA

Supplementary Data are available at NAR Online.

SUPPLEMENTARY DATA
