# Supplementary material for: Chromosome position effects on gene expression in Escherichia coli K-12
Source: Nucleic Acids Res. 2014 Sep 10;42(18):11383–92. doi: 10.1093/nar/gku828 (PMC4191405; doi:10.1093/nar/gku828)
Supplement: SUPPLEMENTARY DATA [file supp_42_18_11383__index.html]

Chromosome position effects on gene expression in Escherichia coli K-12 — Chromosome position effects on gene expression in Escherichia coli K-12 — SUPPLEMENTARY DATA 

# Chromosome position effects on gene expression in *Escherichia coli* K-12

## SUPPLEMENTARY DATA

**Files in this Data Supplement:**

- SUPPLEMENTARY DATA
