# Supplementary material for: Chromosome position effects on gene expression in Escherichia coli K-12
Source: Nucleic Acids Res. 2014 Sep 10;42(18):11383–92. doi: 10.1093/nar/gku828 (PMC4191405; doi:10.1093/nar/gku828)
Supplement: SUPPLEMENTARY DATA [file supp_gku828_nar-02154-h-2014-File008.pdf]

Chromosome Position Effects on Gene Expression in *Escherichia coli* K-12

Jack A. Bryant, Laura E. Sellars, Stephen J. W. Busby, David J. Lee*

SUPPLEMENTARY FIGURES AND TABLES

*Corresponding Author
Institute of Microbiology and Infection,
School of Biosciences,
University of Birmingham,
Edgbaston,
Birmingham,
United Kingdom.
Telephone: +44 (0)121 41 45439
Email: D.lee@bham.ac.uk

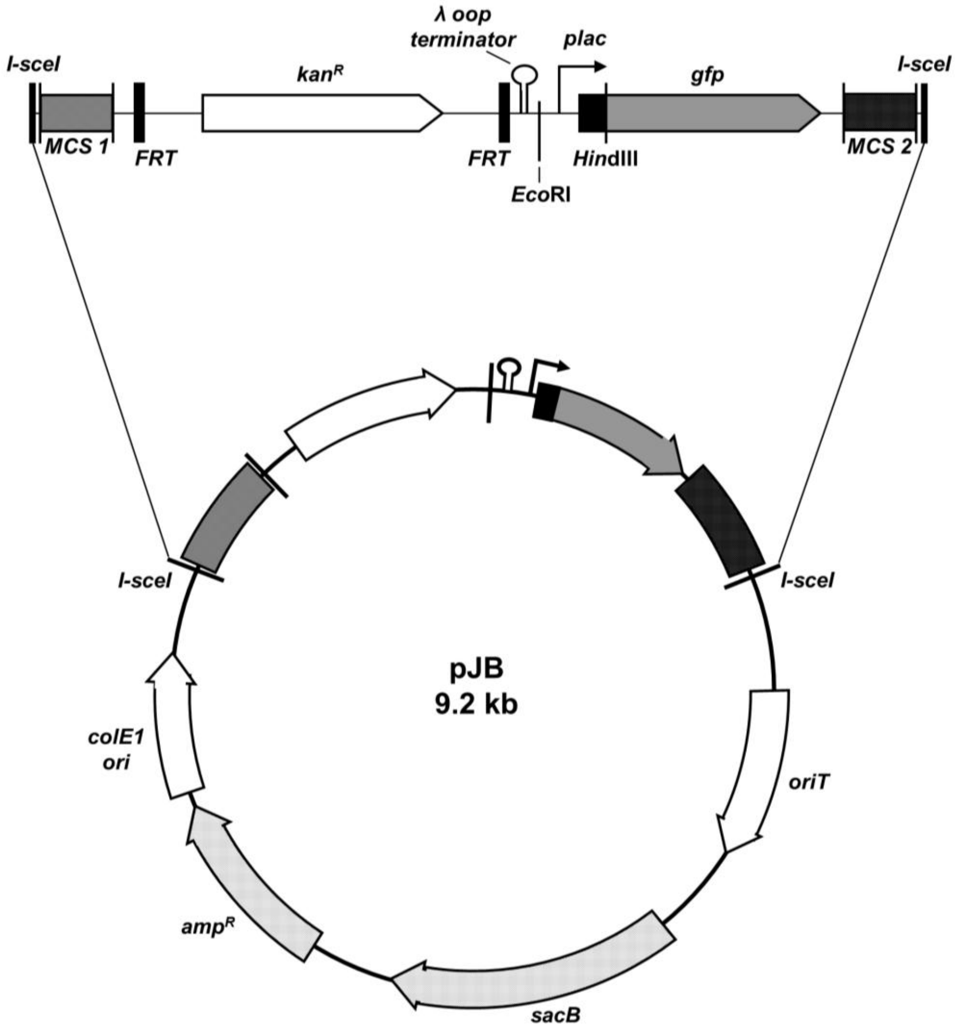

Figure S1. Map of the reporter cassette donor plasmid, pJB. The pJB series of plasmids are derivatives of the pDOC-C gene doctoring plasmid and are used to insert the *lac* promoter*::gfp* reporter cassette at any desired position of the *E. coli* chromosome (1). Approximately 500 bp regions of homology, flanking the desired position of insertion on the chromosome, are cloned into the multiple cloning sites MCS 1 and MCS 2, which flank the reporter cassette. The FRT flanked kanamycin resistance cassette (*kan*^{R}) and λ *oop* transcription terminator (Stem-loop) of pKH5 are included upstream of the fusion to allow selection of recombinants and prevention of transcriptional read-through from neighbouring operons (2). The *kan*^{R} can be excised from the chromosome by Flp recombinase acting at the FRT sites. The plasmid carries the sucrose sensitivity gene (*sacB*) allowing selection against retention of the donor plasmid. The pJB plasmids also carry an ampicillin resistance gene (*ampR*) and origins of replication (*colE1 ori, oriT*).

```
I-SceI           MfeI          XmaI          KpnI   AgeI
tagggataacagggtaatcaattgtatgccccgggttgtaggtaccaactaccggtggctgg
                                FRT
Agctgcttcgaagttcctatactttctagagaataggaacttcggaataggaacttcaagatc
Ccctcacgctgccgcaagcactcagggcgcaagggctgctaaaggaagcggaacacgtagaac
Ttaagggaattgccagctggggcgccctctggtaaggttgggaagccctgcaaagtaaactgg
atggctttcttgccgccaaggatctgatggcgcaggggatcaagatctgatcaagagacagga
                  kanR
tgaggatcgtttcgcATGATTGAACAAGATGGATTGCACGCAGGTTCTCCGGCCGCTTGGGTG
GAGAGGCTATTCGGCTATGACTGGGCACAACAGACAATCGGCTGCTCTGATGCCGCCGTGTTC
CGGCTGTCAGCGCAGGGGCGCCCGGTTCTTTTTGTCAAGACCGACCTGTCCGGTGCCCTGAAT
GAACTGCAGGACGAGGCAGCGCGGCTATCGTGGCTGGCCACGACGGGCGTTCCTTGCGCAGCT
GTGCTCGACGTTGTCACTGAAGCGGGAAGGGACTGGCTGCTATTGGGCGAAGTGCCGGGGCAG
GATCTCCTGTCATCTCACCTTGCTCCTGCCGAGAAAGTATCCATCATGGCTGATGCAATGCGG
CGGCTGCATACGCTTGATCCGGCTACCTGCCCATTCGACCACCAAGCGAAACATCGCATCGAG
CGAGCACGTACTCGGATGGAAGCCGGTCTTGTCGATCAGGATGATCTGGACGAAGAGCATCAG
GGGCTCGCGCCAGCCGAACTGTTCGCCAGGCTCAAGGCGCGCATGCCCGACGGCGAGGATCTC
GTCGTGACCCATGGCGATGCCTGCTTGCCGAATATCATGGTGGAAAATGGCCGCTTTTCTGGA
TTCATCGACTGTGGCCGGCTGGGTGTGGCGGACCGCTATCAGGACATAGCGTTGGCTACCCGT
GATATTGCTGAAGAGCTTGGCGGCGAATGGGCTGACCGCTTCCTCGTGCTTTACGGTATCGCC
GCTCCCGATTCGCAGCGCATCGCCTTCTATCGCCTTCTTGACGAGTTCTTCTGAgcgggactc
Tggggttcgaaatgaccgaccaagcgacgcccaacctgccatcacgagatttcgattccaccg
Ccgccttctatgaaaggttgggcttcggaatcgttttccgggacgccggctggatgatcctcc
agcgcggggatctcatgctggagttctcgcccaccccagcttcaaaagcgctctgaagttcct
          FRT                                   X         X    X
atactttctagagaataggaacttcggaataggaactaaggtaagctagactaagtaattcag
         λ-oop terminator                      EcoRI       LacI O3
aacgctcggttgcccgccgggcgtttttattagatctctcgaggaattcggcagtgagcgca
                   CRP                             -35
acgcaattaatgtgagttagctcactcattaggcaccccaggctttacactttatgcttccgg
      -10         +1      LacI O1                SD         lacZ
ctcgtatgttgtgtggAattgtgagcggataacaatttcacacaggaaacagctATGACCATG
ATTACGGATTCACTGGCCGTCGTTTTACAACGTCGTGACTGGGAAAACCCTGGCGTTACCCAA
              HindIII   gfp
CTTAATCGCCTTaagcttAGCAAGGGCGAGGAGCTGTTCACCGGGGTGGTGCCCATCCTGGTC
GAGCTGGACGGCGACGTAAACGGCcACAAGTTCAGCGTGTCCGGCGAGGGCGAGGGCGATGCC
ACCTACGGCAAGCTGACCCTGAAGTTCATCTGCACCACCGGCAAGCTGCCCGTGCCCTGGCCC
ACCCTCGTGACCACCTTGACCTACGGCGTGCAGTGCTTCGCCCGCTACCCCGACCACATGAAG
CAGCACGACTTCTTCAAGTCCGCCATGCCCGAAGGCTACGTCCAGGAGCGCACCATCTTCTTC
AAGGACGACGGCAACTACAAGACCCGCGCCGAGGTGAAGTTCGAGGGCGACACCCTGGTGAAC
CGCATCGAGCTGAAGGGCATCGACTTCAAGGAGGACGGCAACATCCTGGGGCACAAGCTGGAG
TACAACTACAACAGCCACAAGGTCTATATCACCGCCGACAAGCAGAAGAACGGCATCAAGGTG
AACTTCAAGACCCGCCACAACATCGAGGACGGCAGCGTGCAGCTCGCCGACCACTACCAGCAG
AACACCCCCATCGGCGACGGCCCCGTGCTGCTGCCCGACAACCACTACCTGAGCACCCAGTCC
GCCCTGAGCAAAGACCCCAACGAGAAGCGCGATCACATGGTCCTGCTGGAGTTCGTGACCGCC
                                            X   X
GCCGGGATCACTCTCGGCATGGACGAGCTTTACAAGTAAgtaactaacgaatgtacagccata
SpeI        NheI         SacI          I-SceI
Actagtacttgctagcgcacaagagctctagggataacagggtaat
```

Figure S2. DNA base sequence of the reporter cassette in plasmid pJB. The *I-SceI* DNA target recognition sites are double underlined and labelled. Restriction endonuclease target sites are boxed and labelled. The FLP recognition targets (FRT) DNA sites are shaded grey and labelled. ATG start codon and open reading frames are in capitals with the start and stop codons in bold and underlined. The kanamycin resistance (*kan*^{R}) and *gfp* genes are labelled in uppercase. The *lacZ* start codon and the *lacZ* gene are shown in bold capitals with the Shine-Dalgarno sequence underlined. The promoter -10 and -35 are underlined, with the transcription start site (+1) bold and underlined. The *lacI* operator sequences are boxed and labelled and the DNA site for CRP is double underlined. Additional stop codons in different reading frames were included and are denoted by an X.

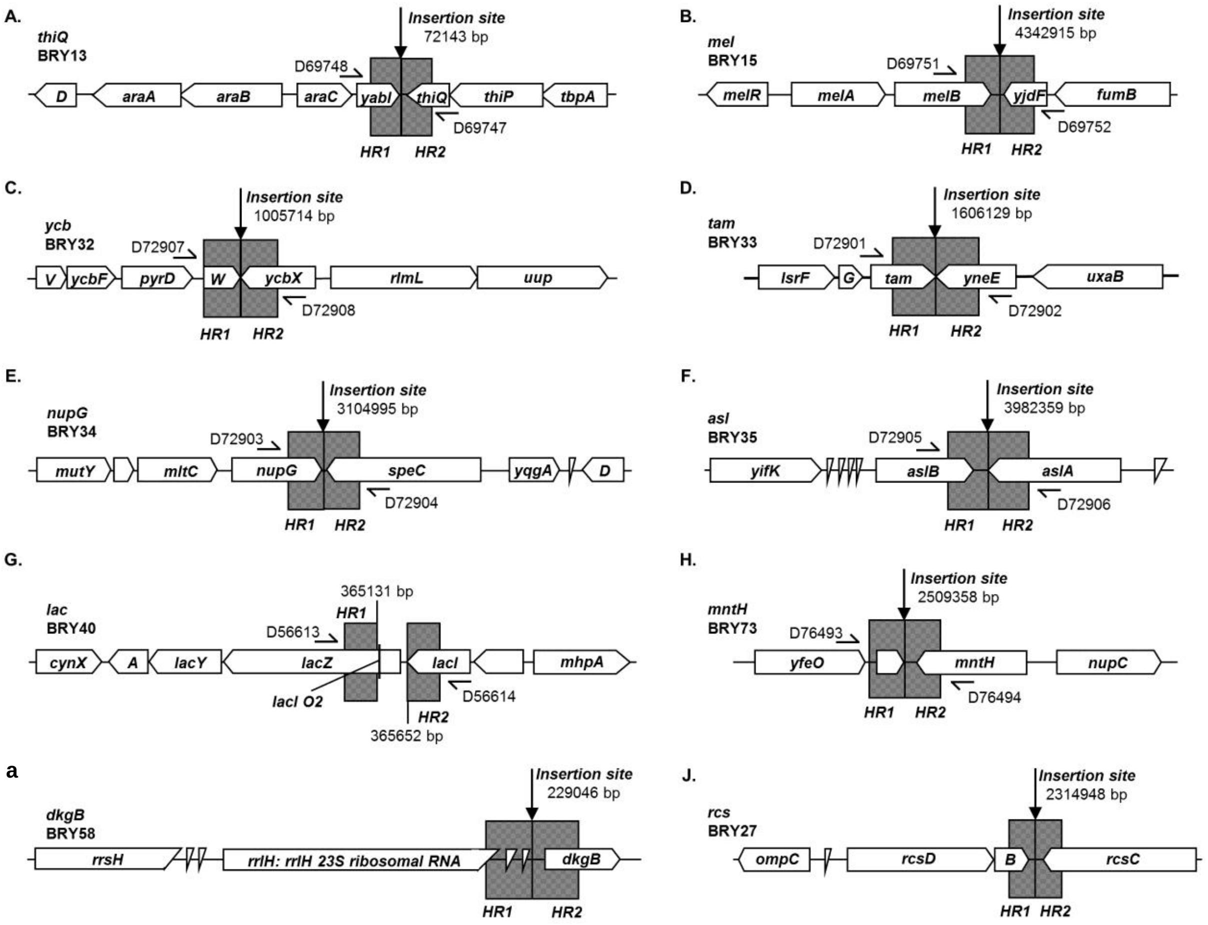

Figure S3. Schematic diagram of gene organisation at reporter cassette insertion sites. Reporter cassette insertion sites (**A** –**J**) with corresponding neighbouring gene name and the name of the derivative BRY strain that was generated. Genes are represented by white block arrows and RNA coding regions represented by white triangles. Insertion sites and the precise base pair position on the chromosome are indicated. Regions of homology, used for insertion of the reporter cassette, are shown as grey shaded boxes, labelled *HR1* and *HR2*.

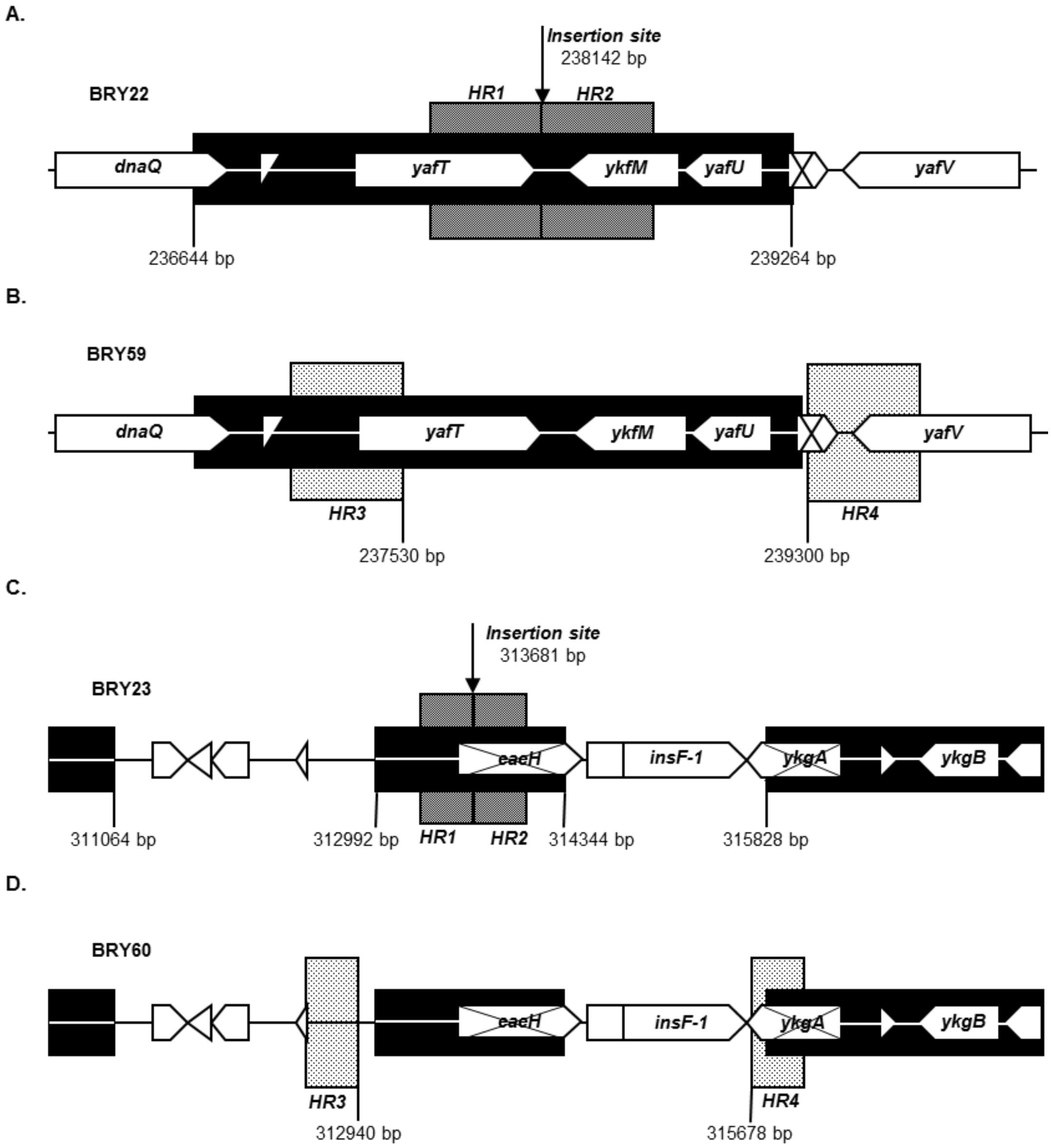

Figure S4. Schematic diagram of *tsEPOD* reporter cassette insertion sites. Organisation of genes at the *yafT* (**A**,**B**) and *eaeH* (**C**,**D**) loci. Genes are represented by white block arrows and white triangles represent RNA coding regions. Crossed boxes represent pseudogenes. Black boxes represent the position of transcriptionally silent extended protein occupancy domains (tsEPOD). The insertion site within the tsEPOD at the *yafT* (**A**) and *eaeH* (**C**) loci are indicated. Homology regions used for recombineering are shown as grey shaded boxes, labelled *HR1* and *HR2*. For tsEPOD replacement at the *yafT* (**B**) and *eaeH* (**D**) loci, homology regions *HR3* and *HR4* (grey shaded boxes) were used for recombineering. The name of the derivative BRY strain that was generated is indicated.

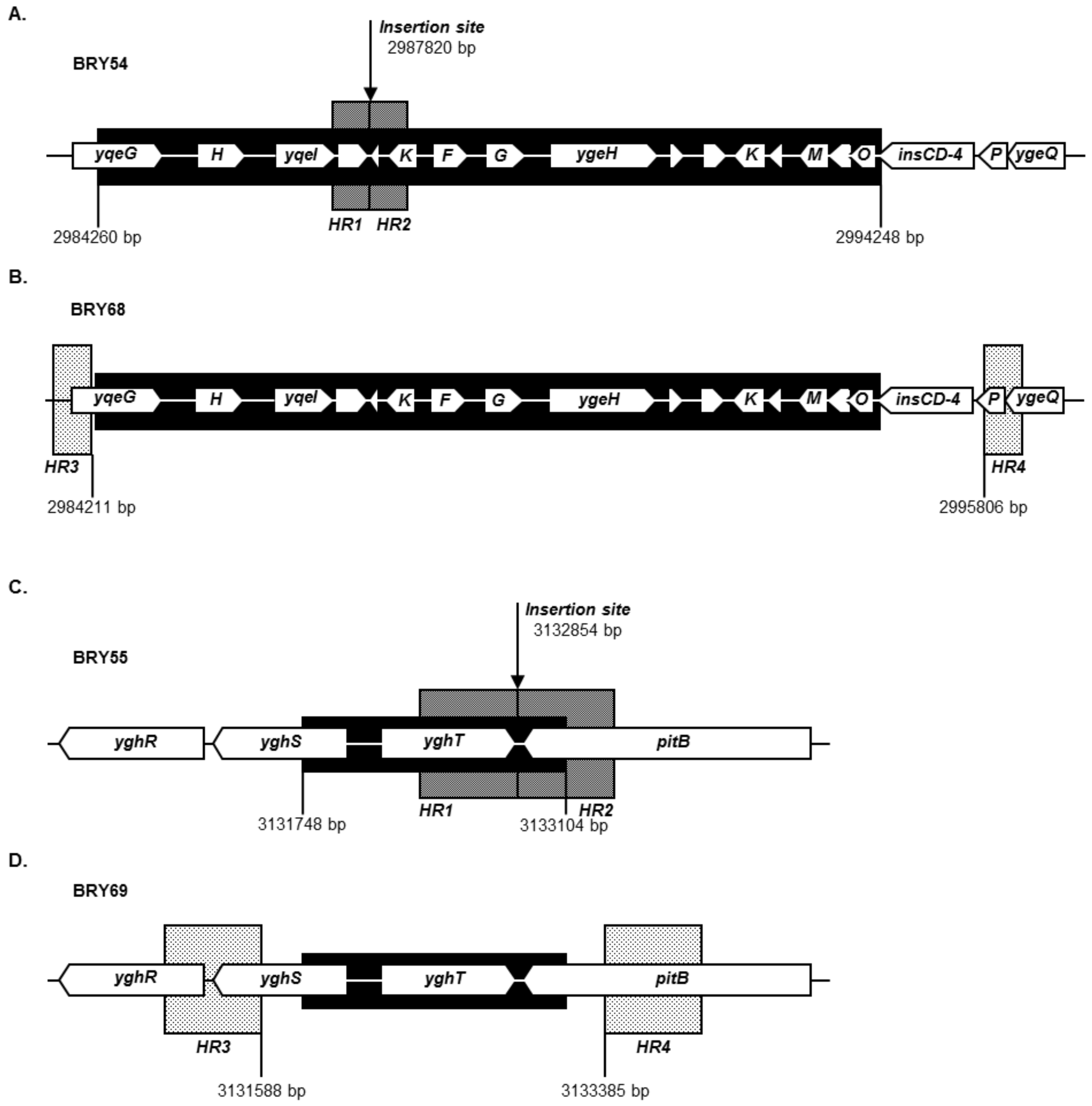

Figure S5. Schematic diagram of *tsEPOD* reporter cassette insertion sites. Organisation of genes at the *yqe* (**A**, **B**) and *pitB* (**C**, **D**) loci. Genes are represented by white block arrows. Black boxes represent the position of transcriptionally silent extended protein occupancy domains (tsEPOD). The insertion site within the tsEPOD at the *yqe* (**A**) and *pitB* (**C**) loci are indicated. Homology regions used for recombineering are shown as grey shaded boxes, labelled *HR1* and *HR2*. For tsEPOD replacement at the *yqe* (**B**) and *pitB* (**D**) loci, homology regions *HR3* and *HR4* (grey shaded boxes) were used for recombineering. The name of the derivative BRY strain that was generated is indicated.

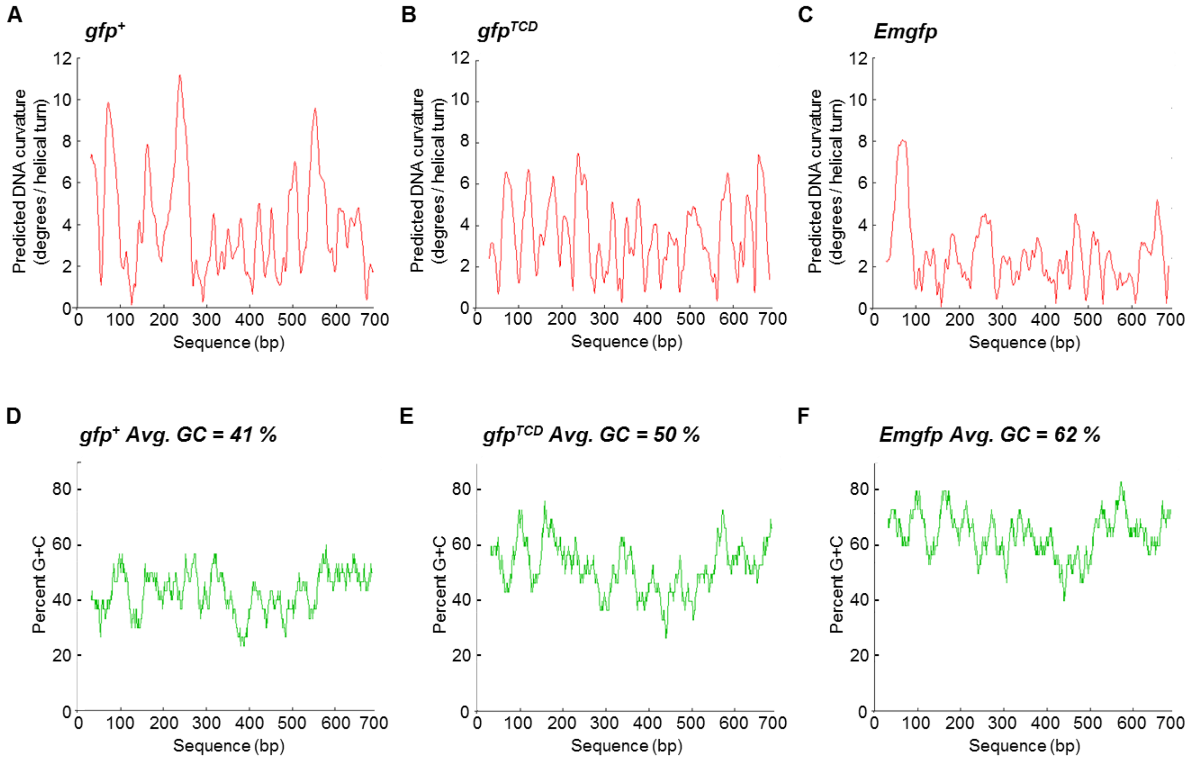

Figure S6. Curvature prediction and GC content of different *gfp* genes. The gene sequence of Emerald GFP (*Emgfp*) was compared with the sequences of *gfp*+ and *gfp*^{TCD}. The derivative *gfp*^{TCD} was previously shown to be less curved and to contain a higher percentage of GC bps compared to *gfp*+ making it less favourable for H-NS binding. Curvature predictions (**A-C**) and G+C content plots (**D-F**) for the *gfp*+, *gfp*^{TCD} and *Emgfp* sequences were created using the Bend.it server (http://hydra.icgeb.trieste.it/dna/bend_form.html), as previously described (3). The curvature parameter used was consensus scale with a curvature window size of 31 and a G+C content window size of 31. The plot type is one dimensional with no smoothing applied.

Table S1. *E. coli* strains used in this study

Strain	Description*	Origin
MG1655	*E. coli* K12 strain	(4)
BRY09	Derivative of MG1655 carrying a *kan* gene and *lac::gfp* fusion at position 72143 bp (*thiQ* locus)	This study
BRY11	Derivative of MG1655 carrying a *kan* gene and *lac::gfp* fusion at position 4342915 bp (*mel* locus)	This study
BRY13	Derivative of BRY09, from which the *kan* gene has been removed. Position 72143 bp (*thiQ1* locus)	This study
BRY15	Derivative of BRY11, from which the *kan* gene has been removed. Position 4342915 bp (*mel1* locus)	This study
BRY17	Derivative of MG1655 carrying a *kan* gene and *lac::gfp* fusion at position 72143 bp in the opposite orientation to that of BRY09. *yabI<kan lac::gfp>thiQ* (*thiQ2* locus)	This study
BRY18	Derivative of MG1655 carrying a *kan* gene and *lac::gfp* fusion at position 238142 bp (*yafT* locus)	This study
BRY19	Derivative of MG1655 carrying a *kan* gene and *lac::gfp* fusion at position 313681 bp (*eaeH* locus)	This study
BRY21	Derivative of BRY17, from which the *kan* gene has been removed. Position 72143 bp (*thiQ2* locus)	This study
BRY22	Derivative of BRY18, from which the *kan* gene has been removed. Position 238142 bp (*yafT* locus)	This study
BRY23	Derivative of BRY19, from which the *kan* gene has been removed. Position 313681 bp (*eaeH* locus)	This study
BRY26	Derivative of MG1655 carrying a *kan* gene and *lac::gfp* fusion at position 2314948 bp (*rcs* locus)	This study
BRY27	Derivative of BRY26, from which the *kan* gene has been removed. Position 2314948 bp (*rcs* locus)	This study
BRY28	Derivative of MG1655 carrying a *kan* gene and *lac::gfp* fusion at position 1005714 bp (*ycb* locus)	This study
BRY29	Derivative of MG1655 carrying a *kan* gene and *lac::gfp* fusion at position 1606129 bp (*tam* locus)	This study
BRY30	Derivative of MG1655 carrying a *kan* gene and *lac::gfp* fusion at position 3104995 bp (*nupG* locus)	This study

Table S1. ***E. coli*** **strains used in this study (continued)**

Strain	Description*	Origin
BRY31	Derivative of MG1655 carrying a *kan* gene and *lac::gfp* fusion at position 3982359 bp (*asl* locus)	This study
BRY32	Derivative of BRY28, from which the *kan* gene has been removed. Position 1005714 bp (*ycb* locus)	This study
BRY33	Derivative of BRY29, from which the *kan* gene has been removed. Position 1606129 bp (*tam* locus)	This study
BRY34	Derivative of BRY30, from which the *kan* gene has been removed. Position 3104995 bp (*nupG* locus)	This study
BRY35	Derivative of BRY31, from which the *kan* gene has been removed. Position 3982359 bp (*asl* locus)	This study
BRY36	Derivative of MG1655 carrying a *kan* gene and *lac::gfp* fusion at position 4342915 bp in the opposite orientation to that of BRY11. *melB<gfp::lac kan>yjdF* (*mel2* locus)	This study
BRY37	Derivative of BRY36, from which the *kan* gene has been removed. Position 4342915 bp (*mel2* locus)	This study
BRY38	Derivative of MG1655, in which the lac promoter and the start of the lacZ gene (including *lacI O2*) was replaced with a *kan* gene and *lac::gfp* fusion (*lac* locus)	This study
BRY39	Derivative of MG1655 carrying a *kan* gene and *lac::gfp* fusion at position 365131-365652 bp in the opposite orientation to that of BRY38. *lacZ<kan lac::gfp>lacI* (*lac2* locus)	This study
BRY40	Derivative of BRY38, from which the *kan* gene has been removed. Position 365131 - 365652 bp (*lac* locus)	This study
BRY41	Derivative of BRY39, from which the *kan* gene has been removed. Position 365131 - 365652 bp (*lac2* locus)	This study
BRY47	Derivative of MG1655 carrying a *kan* gene and *lac::gfp* fusion at position 2987820 bp (*yqe* locus)	This study
BRY48	Derivative of MG1655 carrying a *kan* gene and *lac::gfp* fusion at position 3132854 bp (*pitB* locus)	This study
BRY51	Derivative of MG1655 carrying a *kan* gene and *lac::gfp* fusion at position 229046 bp (*dkgB* locus)	This study
BRY52	Derivative of MG1655 carrying a *kan* gene and *lac::gfp* fusion at position 237530-239300 bp (*ΔyafT* locus)	This study

Table S1. *E. coli* strains used in this study (continued)

Strain	Description*	Origin
BRY53	Derivative of MG1655 carrying a *kan* gene and *lac::gfp* fusion at position 312940-315678 bp (*ΔeaeH* locus)	This study
BRY54	Derivative of BRY47, from which the *kan* gene has been removed. Position 2987820 bp (*yqe* locus)	This study
BRY55	Derivative of BRY48, from which the *kan* gene has been removed. Position 3132854 bp (*pitB* locus)	This study
BRY58	Derivative of BRY51, from which the *kan* gene has been removed. Position 229046 bp (*dkgB* locus)	This study
BRY59	Derivative of BRY52, from which the *kan* gene has been removed. Position 237530 - 239300 bp (*ΔyafT* locus)	This study
BRY60	Derivative of BRY53, from which the *kan* gene has been removed. Position 312940 - 315678 bp (*ΔeaeH* locus)	This study
BRY61	Derivative of MG1655 carrying a *kan* gene and *lac::gfp* fusion at position 2984331 - 2995806 bp (*Δyqe* locus)	This study
BRY62	Derivative of MG1655 carrying a *kan* gene and *lac::gfp* fusion at position 3131588 - 3133385 bp (*ΔpitB* locus)	This study
BRY66	Derivative of MG1655 carrying a *kan* gene and *lac::gfp* fusion at position 2509358 bp (*mntH* locus)	This study
BRY68	Derivative of BRY61, from which the *kan* gene has been removed. Position 2984331 - 2995806 bp (*Δyqe* locus)	This study
BRY69	Derivative of BRY62, from which the *kan* gene has been removed. Position 3131588 - 3133385 bp (*ΔpitB* locus)	This study
BRY73	Derivative of BRY66, from which the *kan* gene has been removed. Position 2509358 bp (*mntH* locus)	This study
BRY75	Lac$^-$ derivative of BRY15, in which the wild type *lac* promoter has been replaced by the cloning site of plasmid pKH3	This study
BRY79	Lac$^-$ derivative of BRY37, in which the wild type *lac* promoter has been replaced by the cloning site of plasmid pKH3	This study

* Position refers to location of the insert in the *E. coli* genome in base pairs (bp) with respect to the coordinate-system origin

Table S2. Oligonucleotides used in this study

Name	Sequence (5' – 3')[1]	Use
D54890	TGAAGGGCAATCAGCTGTTG	Anneals to 3' end of *lacI* gene, upstream of cloning site in pKH3. Used for sequencing inserts in pKH3.
D55668	GAGCGGCGACGATAGTCATG	Anneals downstream of *Sal*I site in pBR322. Used for sequencing inserts in pBR322 during construction of pKH3 and pJB plasmids.
D10520	CCCTGCGGTGCCCCTCAAG	Anneals upstream of *Eco*RI site in pRW50. Used for sequencing and amplification of inserts in this vector.
D56613	CTTGATGTCTCTGACCAGAC	Anneals within the *lacI* sequence outside of the pKH3 homology. Used for screening gene gorging candidates by PCR amplification.
D56614	TTATGCAGCAACGAGACGTC	Anneals within the *lacZ* sequence outside of the pKH3 homology. Used for screening gene gorging candidates by PCR amplification.
D68556	TTTACGTCGCCGTCCAG	Anneals downstream of the start codon of *gfp*. Used for sequencing promoter inserts in pJB plasmid derivatives and BRY strains.
D58793	GGATGTGCTGCAAGG	Sequencing primer to check homology regions inserted between *Nhe*I and *Sac*I. Binds downstream of *I-sceI* site
D58794	TATGCTTCCGGCTCG	Sequencing primer to check homology regions inserted between *Mfe*I and *Xma*I. Binds downstream of *I-sceI* site
D67847	TACGCAATTGATTACCCTGTT<br>ATCCCTAG	Anneals to the *Sce*I site upstream of the *lacI* homology in pKH5 and incorporates an *Mfe*I site. Used with D67848 to create pJB1 by inverse PCR.
D67848	GCTACGACCGGTGGCTGGAG<br>CTGCTTCGAAG	Anneals downstream of the *lacI* homology in pKH5 and incorporates an *Age*I site. Used with D67847 to create pJB1 by inverse PCR.
D67849	AATTGTATGCCCCGGGTTGT<br>AGGTACCAACTA	Complementary to D67876 and encodes the pJB1 multiple cloning site.
D67876	CCGGTAGTTGGTACCTACAA<br>CCCGGGGCATAC	Complementary to D67849 and encodes the pJB1 multiple cloning site.

Table S2. Oligonucleotides used in this study (continued)

Name	Sequence (5' – 3')[1]	Use
D67961	TACGAAGCTTAGCAAGGGCG AGGAGCTG	Anneals to the 5' end of *gfp* in pDEX-G and incorporates a *Hin*dIII site. Used to create pJB2
D67962	TACGGTCGACTTGTATGTGT ACATTCGTTAGTTACTTACTT GTACAGCTCGTCCATG	Anneals to the 3' end of *gfp* in pDEX-G and incorporates two extra stop codons in each reading frame, a *Bsr*GI site and a *Sal*I site. Used to create pJB2 (Figure 2.8).
D68276	CTCGGCATGGACGAGCTTTA CAAGTAAGTAACTAAC	Anneals to the 3' end of *gfp* and incorporates a G→T silent mutation of the *Bsr*GI site. Used to create pJB2ΔB.
D68277	GTTAGTTACTTACTTGTAAAG CTCGTCCATGCCGAG	Anneals to the 3' end of *gfp* and incorporates a G→T silent mutation of the *Bsr*GI site. Used to create pJB2ΔB.
D68085	GTACAGCCATAACTAGTACT TGCTAGCGCACAAGAGCTCT AGGGATAACAGGGTAATG	Complementary to D68086 and encodes the pJB3 multiple cloning site.
D68086	TCGACATTACCCTGTTATCCC TAGAGCTCTTGTGCGCTAGC AAGTACTAGTTATGGCT	Complementary to D68085 and encodes the pJB3 multiple cloning site.
D66948	TATAGAATTCGGGCAGTGAG CGCAACGC	Anneals upstream of O^3 in the *lac* promoter region and incorporates an *Eco*RI site. Used to create the *lac00* and lac fragments.
D68443	GCCGAAGCTTCATAGCTGTT TCCTGTGTG	Anneals to the *lac* promoter translation initiation region and incorporates a *Hin*dIII site downstream of the ATG. Used with D66948 to amplify *lac00* to create pJB4.
D69482	GCCGAAGCTTAAGGCGATTA AGTTGGG	Anneals to *lacZ* to incorporate a *Hin*dIII site immediately downstream of codon 28 to create the *lac* fragment.
Used to amplify homology regions		
D69231	GCCGCAATTGCCGGGATTGA AACTGAACG	Used to amplify the *thiQ* homology region and introduce an *Mfe*I site. Used with D69232. (*thiQ*)
D69232	GCCGCCCGGGCGACGCTTGC CGCGTCTTATC	Used to amplify the *thiQ* homology region and introduce an *Xma*I site. Used with D69231. (*thiQ*)

Table S2. Oligonucleotides used in this study (continued)

Name	Sequence (5' – 3')[1]	Use
D69233	GCCGGAGCTCCTGAACATGC GTTGCATCAAC	Used to amplify the *yabI* homology region and introduce a *Sac*I site. Used with D69234. (*thiQ*)
D69234	GCCGGCTAGCCATCAGGCAA CCCGCAC	Used to amplify the *yabI* homology region and introduce an *Nhe*I site. Used with D69233. (*thiQ*)
D71381	GCCGGAGCTCCCGGGATTGA AACTGAACG	Used to amplify the *thiQ* homology region and introduce a *Sac*I site. Used with D71382. (*thiQ2*)
D71382	GCCGGCTAGCCGACGCTTGC CGCGTCTTATC	Used to amplify the *thiQ* homology region and introduce an *Nhe*I site. Used with D71381. (*thiQ2*)
D71383	GCCGCCCGGGCATCAGGCAA CCCGCAC	Used to amplify the *yabI* homology region and introduce an *Xma*I site. Used with D71384. (*thiQ2*)
D71384	GCCGCAATTGCTGAACATGC GTTGCATCAAC	Used to amplify the *yabI* homology region and introduce an *Mfe*I site. Used with D71383. (*thiQ2*)
D69239	GCCGCAATTGGATCCAGATC CATCTGCTGG	Used to amplify the *melB* homology region and introduce an *Mfe*I site. Used with D69240. (*mel*)
D69240	GCCGCCCGGGTTCCTTATCC GGTCTACAAAATTTG	Used to amplify the *melB* homology region and introduce an *Xma*I site. Used with D69239. (*mel*)
D69241	GCCGGAGCTCCGTTAACGCC GCTGCTCTATACGC	Used to amplify the *yjdF* homology region and introduce a *Sac*I site. Used with D69242. (*mel*)
D69242	GCCGGCTAGCTTCACGCCGC ATCCGGC	Used to amplify the *yjdF* homology region and introduce an *Nhe*I site. Used with D69241. (*mel*)
D72989	GCCGGAGCTCGATCCAGATC CATCTGCTGG	Used to amplify the *melB* homology region and introduce a *Sac*I site. Used with D72876. (*Inverse mel*)
D72876	GCCGGCTAGCTTCCTTATCCG GTCTACAAAATTTG	Used to amplify the *melB* homology region and introduce an *Nhe*I site. Used with D72989. (*Inverse mel*)

Table S2. Oligonucleotides used in this study (continued)

Name	Sequence (5' – 3')[1]	Use
D72988	GCCGCAATTGCGTTAACGCC GCTGCTCTATACGC	Used to amplify the *yjdF* homology region and introduce an *Mfe*I site. Used with D72878. (*Inverse mel*)
D72878	GCCGCCCGGGTTCACGCCGC ATCCGGC	Used to amplify the *yjdF* homology region and introduce an *Xma*I site. Used with D72988. (*Inverse mel*)
D71369	GCCGCAATTGATATTATACT GTTTCCAC	Used to amplify the *yafT* homology region and introduce an *Mfe*I site. Used with D71370. (*yafT*)
D71370	GCCGCCCGGGTCCTGGCGGC AGATATAATTTATTATTG	Used to amplify the *yafT* homology region and introduce an *Xma*I site. Used with D71369. (*yafT*)
D71371	GCCGGCTAGCATTTCTGGCG GCAATAATAC	Used to amplify the *ykfM* homology region and introduce an *Nhe*I site. Used with D71372. (*yafT*)
D71372	GCCGGAGCTCGAGTTAAACC TCTATATTC	Used to amplify the *ykfM* homology region and introduce a *Sac*I site. Used with D71371. (*yafT*)
D71373	GCCGCAATTGATCCATAAAA AATATATTGG	Used to amplify the upstream *eaeH* homology region and introduce an *Mfe*I site. Used with D71374. (*eaeH*)
D71374	GCCGCCCGGGGAAAAAGAA CCTGAACAGAG	Used to amplify the upstream *eaeH* homology region and introduce an *Xma*I site. Used with D71373. (*eaeH*)
D71375	GCCGGCTAGCCACTCGCTGT CACCTTTACC	Used to amplify the downstream *eaeH* homology region and introduce an *Nhe*I site. Used with D71376. (*eaeH*)
D71376	GCCGGAGCTCGCACCAACAC CAATGCG	Used to amplify the downstream *eaeH* homology region and introduce a *Sac*I site. Used with D71375. (*eaeH*)
D71881	GGACCAATTGATTGTTCTGA CTATGAACAACAACC	Used to amplify the *rcsB* homology region and introduce an *Mfe*I site. Used with D71882. (*rcs*)
D71882	GGACCCCGGGATGGGAATCG TAGGCCG	Used to amplify the *rcsB* homology region and introduce an *Xma*I site. Used with D71881. (*rcs*)

Table S2. Oligonucleotides used in this study (continued)

Name	Sequence (5' – 3')[1]	Use
D71883	GGACGCTAGCTATTTCAACAAATTACATTAAAGTAGG	Used to amplify the rcsC homology region and introduce an *Nhe*I site. Used with D71884. (*rcs*)
D71884	GGACGAGCTCGCGGTCAGCGATAATGAC	Used to amplify the *rcsC* homology region and introduce a *Sac*I site. Used with D71883. (*rcs*)
D72684	CGGCCAATTGAAGAGCACGATCGAATGATG	Used to amplify the *ycbW* homology region and introduce an *Mfe*I site. Used with D72685. (*ycb*)
D72685	CGGCCCCGGGTTAGACTGCCTGTTCGAGG	Used to amplify the *ycbW* homology region and introduce an *Xma*I site. Used with D72684. (*ycb*)
D72686	CGGCGCTAGCCTAACGCGCCAACTTAAG	Used to amplify the *ycbX* homology region and introduce an *Nhe*I site. Used with D72687. (*ycb*)
D72687	CGGCGAGCTCCTAGCCGCTGTATTTTCACC	Used to amplify the *ycbX* homology region and introduce a *Sac*I site. Used with D72686. (*ycb*)
D72688	CGGCCAATTGCTGGCAACCGGTACAGG	Used to amplify the *tam* homology region and introduce an *Mfe*I site. Used with D72689. (*tam*)
D72689	CGGCCCCGGGTTTACTCCATACGCCGGG	Used to amplify the *tam* homology region and introduce an *Xma*I site. Used with D72688. (*tam*)
D72690	CGGCGCTAGCTATCACGTCAGCTGGTAATG	Used to amplify the *yneE* homology region and introduce an *Nhe*I site. Used with D72691. (*tam*)
D72691	CGGCGAGCTCTATCTCAAGACTGAAGATCTTCAG	Used to amplify the *yneE* homology region and introduce a *Sac*I site. Used with D72690. (*tam*)
D72692	CGGCCAATTGCAATCATCATGTCGATTTC	Used to amplify the *nupG* homology region and introduce an *Mfe*I site. Used with D72693. (*nupG*)
D72693	CGGCCCCGGGTAATTAGTGGCTAACCGTC	Used to amplify the *nupG* homology region and introduce an *Xma*I site. Used with D72692. (*nupG*)

Table S2. Oligonucleotides used in this study (continued)

Name	Sequence (5' – 3')[1]	Use
D72694	CGGCGCTAGCCGCAAAGAAAAACGGGTC	Used to amplify the *speC* homology region and introduce an *Nhe*I site. Used with D72695. (*nupG*)
D72695	CGGCGAGCTCGTGTTGCCGAGCGTTTATAAC	Used to amplify the *speC* homology region and introduce a *Sac*I site. Used with D72694. (*nupG*)
D72695	CGGCGAGCTCGTGTTGCCGAGCGTTTATAAC	Used to amplify the *speC* homology region and introduce a *Sac*I site. Used with D72694. (*nupG*)
D72696	CAAACAATTGCAGAAATGATCG	Used to amplify the *aslB* homology region and introduce an *Mfe*I site. Used with D72697. (*asl*)
D72697	CGGCCCCGGGGAGATCTGCCTTTGCCG	Used to amplify the *aslB* homology region and introduce an *Xma*I site. Used with D72696. (*asl*)
D72698	CGGCGCTAGCAGCGATAGCGCCGGCTTAG	Used to amplify the *aslA* homology region and introduce an *Nhe*I site. Used with D72699. (*asl*)
D72699	CGGCGAGCTCGGAAAGGGATGATCCAACCG	Used to amplify the *aslA* homology region and introduce a *Sac*I site. Used with D72698. (*asl*)
D72369	GCCGGCTAGCCTGGCTACAGGAAGGCC	Used to amplify the *lacZ* homology region and introduce an *Nhe*I site. Used with D72370. (*lac*)
D72370	GCCGGAGCTCGTTTCGGGTTTTCGACG	Used to amplify the *lacZ* homology region and introduce a *Sac*I site. Used with D72369. (*lac*)
D68504	TACGCAATTGCCATGAAGACGGTACGCG	Used to amplify the *lacI* homology region and introduce an *Mfe*I site. Used with D68505. (*lac*)
D68505	TACGCCCGGGTCACTGCCCGCTTTCCAG	Used to amplify the *lacI* homology region and introduce an *Xma*I site. Used with D68504. (*lac*)
D72371	GGACCAATGGTTTCGGGTTTTCGACG	Used to amplify the *inverse lacZ* homology region and introduce an *Mfe*I site. Used with D72372. (*Inverse lac*)

Table S2. Oligonucleotides used in this study (continued)

Name	Sequence (5' – 3')[1]	Use
D72372	GGACCCCGGGCTGGCTACAG GAAGGCC	Used to amplify the *inverse lacZ* homology region and introduce an *Xma*I site. Used with D72371. (*Inverse lac*)
D71377	TACGGAGCTCCCATGAAGAC GGTACGCG	Used to amplify the *inverse lacI* homology region and introduce a *Sac*I site. Used with D71378. (*Inverse lac*)
D71378	TACGGCTAGCTCACTGCCCG CTTTCCAG	Used to amplify the *inverse lacI* homology region and introduce an *Nhe*I site. Used with D71377. (*Inverse lac*)
D68765	GGACGCTAGCATGACCATGA TTACGGATTC	Used to amplify the *lacZ (+O_2)* homology region and introduce an *Nhe*I site. Used with D68766. (*lac +O_2*)
D68766	GGACGAGCTCCACATATCCT GATCTTCCAG	Used to amplify the *lacZ (+O_2)* homology region and introduce a *Sac*I site. Used with D68765. (*lac +O_2*)
D75746	GCTCAATTGATGAGGCTTAT CTGACGC	Used to amplify the *mntH* homology region and introduce an *Mfe*I site. Used with D75747. (*mntH*)
D75747	GCTCCCGGGGCCAATGGAGC ACAATGC	Used to amplify the *mntH* homology region and introduce an *Xma*I site. Used with D75746. (*mntH*)
D75748	GCTGCTAGCGGACGCGTTTA ATGGCG	Used to amplify the *ypeC* homology region and introduce an *Nhe*I site. Used with D75749. (*mntH*)
D75749	GCTGAGCTCGTGCTGGTGGT AACACG	Used to amplify the *ypeC* homology region and introduce a *Sac*I site. Used with D75748. (*mntH*)
D74762	CGGCCAATTGCCTGACTCCTT GAGAGTCC	Used to amplify the *aspU* homology region and introduce an *Mfe*I site. Used with D74763. (*dkgB*)
D74763	CGGCCCCGGGGAAAAAAAA CCTCGAGCG	Used to amplify the *aspU* homology region and introduce an *Xma*I site. Used with D74762. (*dkgB*)
D74764	CGGCGCTAGCGTCTGTATAT CTATTATTGCCAG	Used to amplify the *dkgB* homology region and introduce an *Nhe*I site. Used with D74765. (*dkgB*)

Table S2. Oligonucleotides used in this study (continued)

Name	Sequence (5' – 3')[1]	Use
D74765	CGGCGAGCTCAAATACCGATCTCACGC	Used to amplify the *dkgB* homology region and introduce a *Sac*I site. Used with D74764. (*dkgB*)
D74746	CGGCCAATTGGTGATTGATTATAAAAAAAAC	Used to amplify the *yqeJ* homology region and introduce an *Mfe*I site. Used with D74747. (*yqe*)
D74747	CGGCCCCGGGATGTATTTTAGTTTACCTTGC	Used to amplify the *yqeJ* homology region and introduce an *Xma*I site. Used with D74746. (*yqe*)
D74748	CGGCGCTAGCAACAAAAAGATGGAACCTCG	Used to amplify the *yqeL* homology region and introduce an *Nhe*I site. Used with D74749. (*yqe*)
D74749	CGGCGAGCTCCACGAAAAAGAAACCAAGG	Used to amplify the *yqeL* homology region and introduce a *Sac*I site. Used with D74748. (*yqe*)
D74750	CGGCCAATTGCAAAAAAATTGCCTGGACCAG	Used to amplify the *yghT* homology region and introduce an *Mfe*I site. Used with D74751. (*pitB*)
D74751	CGGCCCCGGGCTGGCGATCGGGCATTTC	Used to amplify the *yghT* homology region and introduce an *Xma*I site. Used with D74750. (*pitB*)
D74752	CGGCGCTAGCGACCGGGCATTTTCAGG	Used to amplify the *pitB* homology region and introduce an *Nhe*I site. Used with D74753. (*pitB*)
D74753	CGGCGAGCTCGAAACTGCCAGGCGTCAG	Used to amplify the *pitB* homology region and introduce a *Sac*I site. Used with D74752. (*pitB*)
D75191	GGACCAATTGGATTTGCTGTACTTTATTC	Used to amplify the upstream *ΔeaeH* homology region and introduce an *Mfe*I site. Used with D75192. (*ΔeaeH*)
D75192	GGACCCCGGGATATCTATTATTCTCCCG	Used to amplify the upstream *ΔeaeH* homology region and introduce an *Xma*I site. Used with D71373. (*ΔeaeH*)
D76797	GCCGGCTAGCGGCCTGTGTCCATATTACG	Used to amplify the downstream homology *ΔeaeH* region and introduce an *Nhe*I site. Used with D71376. (*ΔeaeH*)

Table S2. Oligonucleotides used in this study (continued)

Name	Sequence (5' – 3')[1]	Use
D76798	GCCGGAGCTCCACCGCGAAATCTATGC	Used to amplify the downstream *ΔeaeH* homology region and introduce a *Sac*I site. Used with D71375. (*ΔeaeH*)
D75195	GGACCAATTGTGAGTTCAGAGAGCCGC	Used to amplify the upstream *ΔyafT* homology region and introduce an *Mfe*I site. Used with D75196. (*ΔyafT*)
D75196	GGACCCCGGGATCCTCTTCGGATACGG	Used to amplify the upstream *ΔyafT* homology region and introduce an *Xma*I site. Used with D75195. (*ΔyafT*)
D75197	GCCGGCTAGCGTTAAGACGTAAGATGCG	Used to amplify the downstream homology *ΔyafT* region and introduce an *Nhe*I site. Used with D75198. (*ΔyafT*)
D75198	GCCGGAGCTCTGCTACGACTTACGTTTTC	Used to amplify the downstream *ΔyafT* homology region and introduce a *Sac*I site. Used with D75197. (*ΔyafT*)
D76801	GGACCAATTGAAATATGCCTGAGCAGC	Used to amplify the upstream *Δyqe* homology region and introduce an *Mfe*I site. Used with D76802. (*Δyqe*)
D76802	GGACCCCGGGTTCCGTAAGTGAGTTGG	Used to amplify the upstream *Δyqe* homology region and introduce an *Xma*I site. Used with D76801. (*Δyqe*)
D76803	GCCGGCTAGCTTGTCTGGAGATTCAGG	Used to amplify the downstream homology *Δyqe* region and introduce an *Nhe*I site. Used with D76804. (*Δyqe*)
D76804	GCCGGAGCTCCAAGCAATCAACACTGG	Used to amplify the downstream *Δyqe* homology region and introduce a *Sac*I site. Used with D76803. (*Δyqe*)
D76805	GGACCAATTGATCGTTTGATTTTGTCG	Used to amplify the upstream *ΔpitB* homology region and introduce an *Mfe*I site. Used with D76806. (*ΔpitB*)
D76806	GGACCCCGGGAAAAATGTTAAGGCAGC	Used to amplify the upstream *ΔpitB* homology region and introduce an *Xma*I site. Used with D76805. (*ΔpitB*)
D76807	GTTTCGCTAGCTTCGCGG	Used to amplify the downstream homology *ΔpitB* region and introduce an *Nhe*I site. Used with D76808. (*ΔpitB*)

Table S2. Oligonucleotides used in this study (continued)

Name	Sequence (5' – 3')[1]	Use
D76808	GCCGGAGCTCAATGCTGGTA CTGGTGG	Used to amplify the downstream *ΔpitB* homology region and introduce a *Sac*I site. Used with D76807. (*ΔpitB*)
Used to screen candidates		
D54890	TGAAGGGCAATCAGCTGTTG	Anneals to 3' end of *lacI* gene, upstream of cloning site in pKH3. Used for sequencing inserts in pKH3.
D55668	GAGCGGCGACGATAGTCATG	Anneals downstream of SalI site in pBR322. Used for sequencing inserts in pBR322 during construction of pKH3.
D10520	CCCTGCGGTGCCCCTCAAG	Anneals upstream of *Eco*RI site in pRW50. Used for sequencing and amplification of inserts in this vector.
D56613	CTTGATGTCTCTGACCAGAC	Anneals within the *lacI* sequence outside of the pKH3 homology. Used for screening gene gorging candidates by PCR amplification.
D56614	TTATGCAGCAACGAGACGTC	Anneals within the *lacZ* sequence outside of the pKH3 homology. Used for screening gene gorging candidates by PCR amplification.
D68556	TTTACGTCGCCGTCCAG	Anneals downstream of the start codon of *gfp*. Used for sequencing promoter inserts in pJB3 derivatives and BRY strains.
D69747	GTCGCACAGAACATCGG	Anneals to *thiQ* gene outside of the homology regions used. Used for PCR screening with D69748. (*thiQ*)
D69748	TCGCTGGTCATTTCTGAAG	Anneals to *yabI* gene outside of the homology regions used. Used for PCR screening with D69747. (*thiQ*)
D69751	TATCGCCTCAATGGTGACA	Anneals to *melB* gene outside of the homology regions used. Used for PCR screening with D69752. (*mel*)
D69752	TTGCCACCGCCAGAC	Anneals to *yjdF* gene outside of the homology regions used. Used for PCR screening with D69751. (*mel*)
D71835	GGAAACCATTATGCAGGAGG	Anneals to *yafT* gene outside of the homology regions used. (*yafT*)

Table S2. Oligonucleotides used in this study (continued)

Name	Sequence (5' – 3')[1]	Use
D71836	TTTCCCTGCATTCAATGC	Anneals to *ykfM* gene outside of the homology regions used. Used for PCR screening with D71835. (*yafT*)
D71837	AATTTACAGTCCGATGAAGG	Anneals to *eaeH* gene upstream of the homology regions used. Used for PCR screening with D71838. (*eaeH*)
D71838	TTTCAGATAATCGCGCC	Anneals to *eaeH* gene upstream of the homology regions used. Used for PCR screening with D71837. (*eaeH*)
D72064	GCGCCATTTCCCAAGCC	Anneals to *rcsB* gene upstream of the homology regions used. Used for PCR screening with D72065. (*rcs*)
D72065	CGCTCTGCCGTCAACGG	Anneals to *rcsC* gene upstream of the homology regions used. Used for PCR screening with D72064. (*rcs*)
D72901	GTGCTTTGCCAGACTGC	Anneals to *tam* gene outside of the homology regions used. Used for PCR screening with D72902. (*tam*)
D72902	TGACATTACGCAAACAGCC	Anneals to *yneE* gene outside of the homology regions used. Used for PCR screening with D72901. (*tam*)
D72903	ATCCGATGTTTGCCAGC	Anneals to *nupG* gene outside of the homology regions used. Used for PCR screening with D72904. (*nupG*)
D72904	GCATAATGAACAGCATATTG AGG	Anneals to *speC* gene outside of the homology regions used. Used for PCR screening with D72903. (*nupG*)
D72905	CACTATGTTTATCCGCAATAT CG	Anneals to *aslB* gene outside of the homology regions used. Used for PCR screening with D72906. (*asl*)
D72906	TCGCGTACCGACTTTCG	Anneals to *aslC* gene outside of the homology regions used. Used for PCR screening with D72905. (*asl*)
D72907	GGCAGATGCGAATTAAACC	Anneals to *ycbW* gene outside of the homology regions used. Used for PCR screening with D72908. (*ycb*)

Table S2. Oligonucleotides used in this study (continued)

Name	Sequence (5' – 3')[1]	Use
D72908	TCGCATTGGTGATGTGG	Anneals to *ycbX* gene outside of the homology regions used. Used for PCR screening with D72907. (*ycb*)
D76493	GCTACAGCTGCGGCGGC	Anneals to *mntH* gene outside of the homology regions used. Used for PCR screening with D76494. (*mntH*)
D76494	GCGGCAATAACCGTTTCTTG CG	Anneals to *ypeC* gene outside of the homology regions used. Used for PCR screening with D76493. (*mntH*)
D75808	AGTGGACGCATCACTGG	Anneals to *aspU* gene outside of the homology regions used. Used for PCR screening with D75809. (*dkgB*)
D75809	CCTCATCTTTCAGGGCC	Anneals to *dkgB* gene outside of the homology regions used. Used for PCR screening with D75808. (*dkgB*)
D75800	AGCGCATTTGTCATAGG	Anneals to *yqeJ* gene outside of the homology regions used. Used for PCR screening with D75801. (*yqe*)
D75801	TTTCTCTCATTTGATTAGAGC	Anneals to *yqeL* gene outside of the homology regions used. Used for PCR screening with D75800. (*yqe*)
D75802	ACACCTCCATTAATTGC	Anneals to *yghT* gene outside of the homology regions used. Used for PCR screening with D75803. (*pitB*)
D75803	ACACTACCTGCAACAGC	Anneals to *pitB* gene outside of the homology regions used. Used for PCR screening with D75802. (*pitB*)
D75810	ACCTGACAGCGTGTATTCC	Check primer for *ΔeaeH*, which anneals outside of the homology regions used. Used for PCR screening. (*ΔeaeH*)
D75812	CATTACAAACGGAGTCTGG	Check primer for *ΔyafT*, which anneals upstream of the homology regions used. Used for PCR screening with D75813. (*ΔyafT*)
D75813	AAAACCGTTGACGAAGG	Check primer for *ΔyafT*, which anneals downstream of the homology regions used. Used for PCR screening with D75812. (*ΔyafT*)

Table S2. Oligonucleotides used in this study (continued)

Name	Sequence (5' – 3')[1]	Use
D77712	GTCTTTCATCACAACCTCG	Check primer for *Δyqe*, which anneals upstream of the homology regions used. Used for PCR screening with D77713. (*Δyqe*)
D77713	GGTCTTTACCTTGATCTCC	Check primer for *Δyqe*, which anneals downstream of the homology regions used. Used for PCR screening with D77712. (*Δyqe*)
D77714	GGAGATTTGGTTTTCATGC	Check primer for *ΔpitB*, which anneals upstream of the homology regions used. Used for PCR screening with D77715. (*ΔpitB*)
D77715	TTGCGCTGATTGTTTCC	Check primer for *ΔpitB*, which anneals downstream of the homology regions used. Used for PCR screening with D77714. (*ΔpitB*)
Used for qPCR		
bglB upstream	GTCGTTACACGCGCCATTCAC	Anneals to the *bglB* gene. Used with *bglB* downstream as an internal control for qPCR
bglB downstream	AACCAGCCCACCGAGAAGC	Anneals to the *bglB* gene. Used with *bglB* upstream as an internal control for qPCR
gfp upstream	GCGACGTAAACGGCCACAAG	Anneals to the *gfp* gene. Used with *gfp* downstream for qPCR
gfp downstream	GTAGCGGGCGAAGCACTG	Anneals to the *gfp* gene. Used with *gfp* upstream for qPCR

[1] Target sites for restriction endonucleases are underlined.

References

1. Lee, D.J., Bingle, L.E., Heurlier, K., Pallen, M.J., Penn, C.W., Busby, S.J. and Hobman, J.L. (2009) Gene doctoring: a method for recombineering in laboratory and pathogenic Escherichia coli strains. *BMC Microbiol*, **9**, 252.
2. Hollands, K. (2009), University of Birmingham, Birmingham.
3. Corcoran, C.P., Cameron, A.D. and Dorman, C.J. (2010) H-NS silences gfp, the green fluorescent protein gene: gfpTCD is a genetically Remastered gfp gene with reduced susceptibility to H-NS-mediated transcription silencing and with enhanced translation. *J Bacteriol*, **192**, 4790-4793.
4. Blattner, F.R., Plunkett, G., 3rd, Bloch, C.A., Perna, N.T., Burland, V., Riley, M., Collado-Vides, J., Glasner, J.D., Rode, C.K., Mayhew, G.F. *et al.* (1997) The complete genome sequence of Escherichia coli K-12. *Science*, **277**, 1453-1462.
